# Supplementary material for: Flexible neural population dynamics govern the speed and stability of sensory encoding in mouse visual cortex
Source: Nat Commun. 2024 Jul 30;15:6415. doi: 10.1038/s41467-024-50563-y (PMC11289260; doi:10.1038/s41467-024-50563-y)
Supplement: Supplementary file 1 — Supplementary Information [file 41467_2024_50563_MOESM1_ESM.pdf]

Supplementary Figures

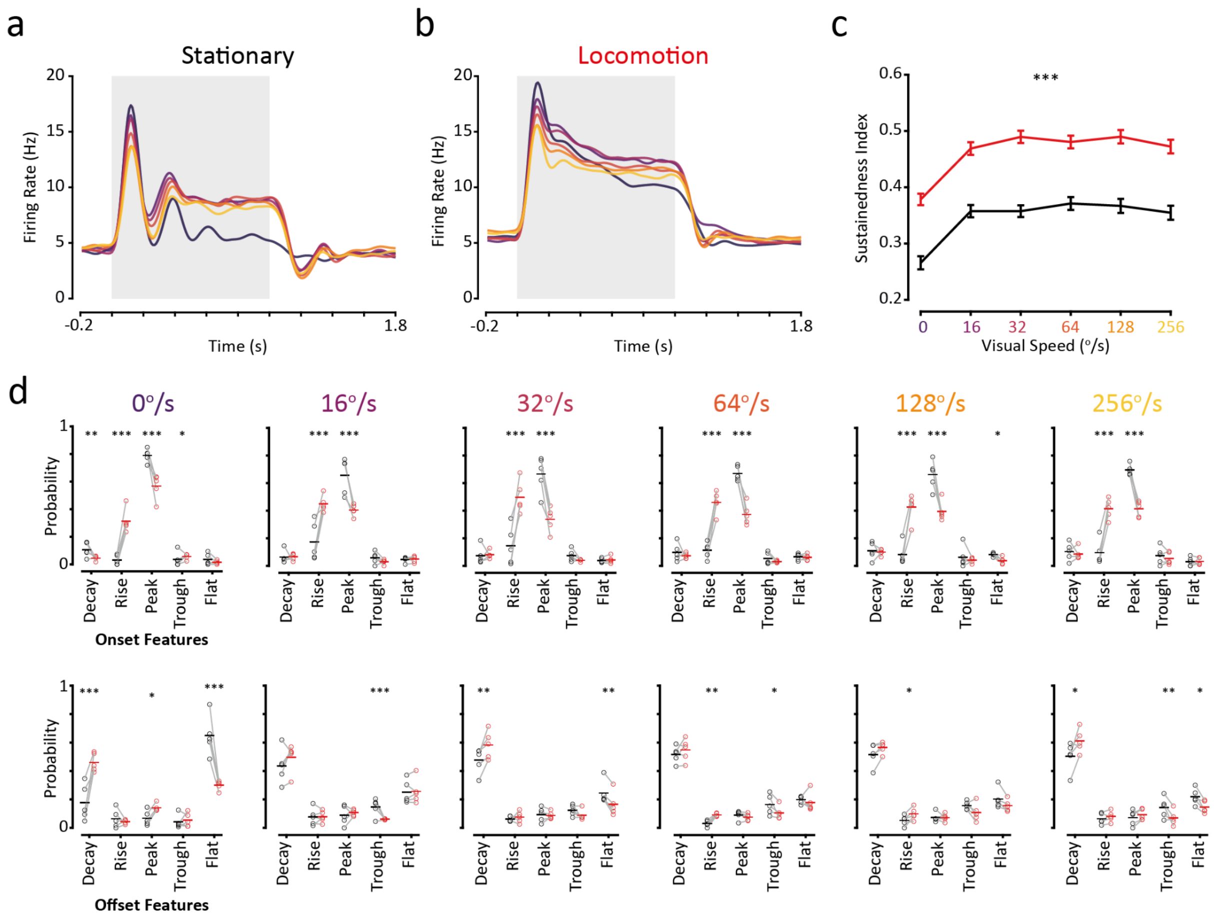

Supplementary Figure 1: Effects of behavioural state on single-neuron responses to different stimulus speeds

a) Mean reliable PSTHs for each of the six visual speeds presented in stationary trials. Shaded regions indicate mean ± SEM across subjects (n=5). **b)** Same as (a) for locomotion trials. **c)** Mean sustainedness index of reliable responses for each of the six visual speeds presented in stationary (black) and locomotion (red) trials. Errorbars indicate mean ± SEM across subjects (n=5). LME model analysis, two-sided effect of behavioural state. All speeds *** *p<0.001.* **d)** Proportions of reliable responses classified with different onset (top panels) and offset (bottom panels) features, for each of the six visual speeds presented. Individual data points are paired proportions of responses from each session for stationary (black) and locomotion (red) trials. Horizontal lines represent the fraction of all reliable responses with a given feature. GLME model analysis, two-sided effect of behavioural state. Adjustments were not made for multiple comparisons.

** p<0.05; ** p<0.01; *** p<0.001.*

Source data are provided as a Source Data file.

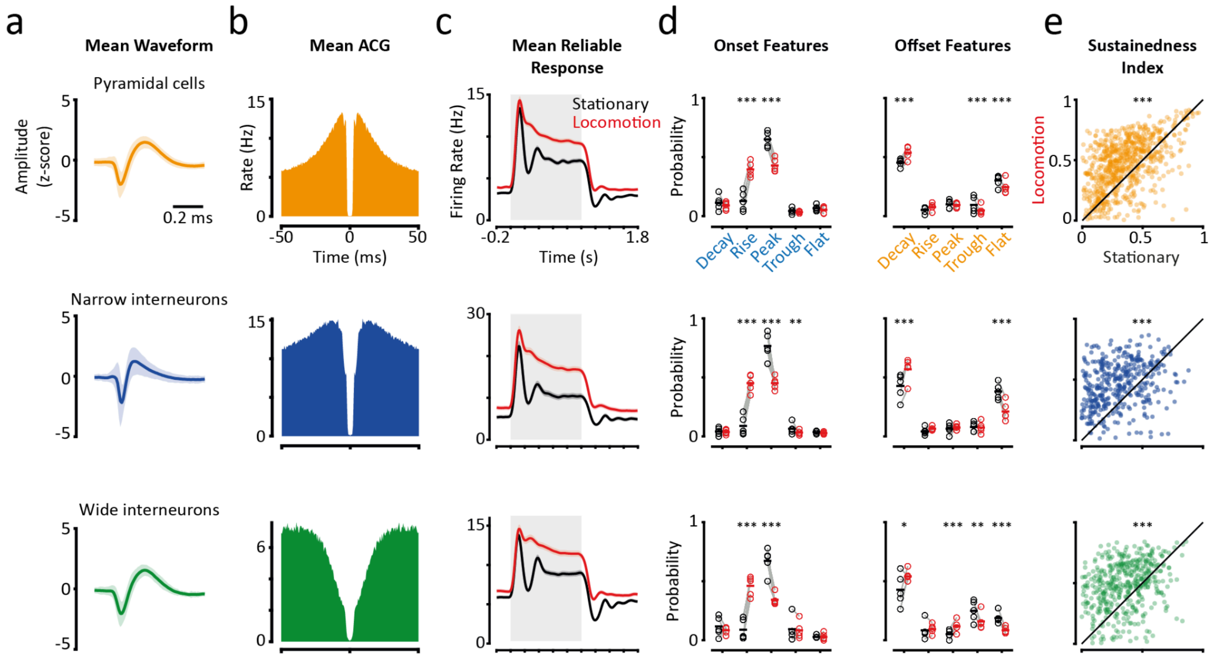

Supplementary Figure 2: Effects of behavioural state on single-neuron responses for different physiologically-defined cell-types

a) Mean spike waveforms for each classified cell-type (coloured lines) with example individual cell spike waveforms shown as thin grey lines. Shaded regions indicate mean ± SEM across cells (n=959 pyramidal cells, n=370 narrow interneurons, n=254 wide interneurons). **b)** Mean autocorrelogram (ACG) for each cell-type. **c)** Mean reliable PSTHs from stationary (black) and locomotion (red) trials for each cell-type. **d)** Proportions of reliable responses classified with different onset (left panels) and offset (right panels) features for each cell-type. Individual data points are paired proportions of responses from each session for stationary (black) and locomotion (red) trials. Horizontal lines represent the fraction of all reliable responses with a given feature. GLME model analysis, two-sided effect of behavioural state. ** $p<0.05$; ** $p<0.01$; *** $p<0.001$.* **e)** Scatter plot of sustainedness index of reliable responses in stationary and locomotion trials, for each cell-type. LME model analysis, two-sided effect of behavioural state. *** *$p<0.001$.*

Source data are provided as a Source Data file.

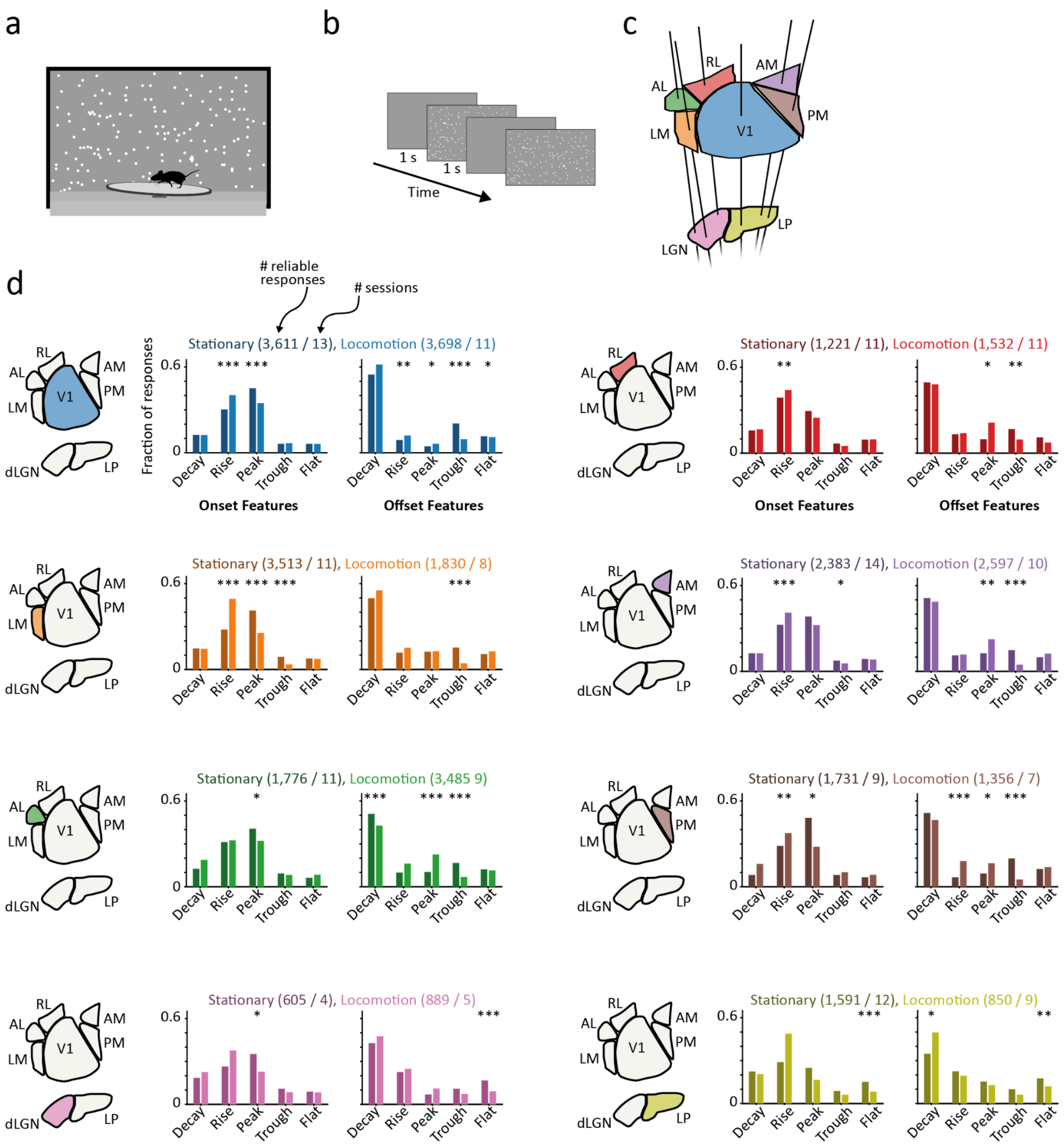

Supplementary Figure 3: Effects of behavioural state on single-neuron responses in different visual areas

We analysed single-neuron responses to moving dot fields throughout the mouse visual cortex and thalamus during stationary and locomotion states using the Allen Institute's 'Visual Coding'[55] dataset.

a) Moving dot field stimuli were presented whilst mice were free to locomote on a disc. **b)** Stimuli had one second duration with a one second grey screen intertrial interval. **c)** Illustration of probe insertions through 6 cortical visual areas and two thalamic visual areas. Image based on[55]. **d)** Analysis of visual areas. Left panel: schematic location of visual area in left hemisphere. Centre and right panels: proportions of reliable responses classified with different onset (centre panels) and offset (right panels) features for each visual area. Individual data points are (paired) proportions of responses from each session for stationary (black) and locomotion (red) trials. Horizontal lines represent the fraction of all reliable

responses with a given feature. Text above plots shows the number of reliable responses analysed in each visual area, separately for sessions with stationary or locomotion trials. GLME model analysis, two-sided effect of behavioural state.

** p<0.05; ** p<0.01; *** p<0.001.*

Source data are provided as a Source Data file.

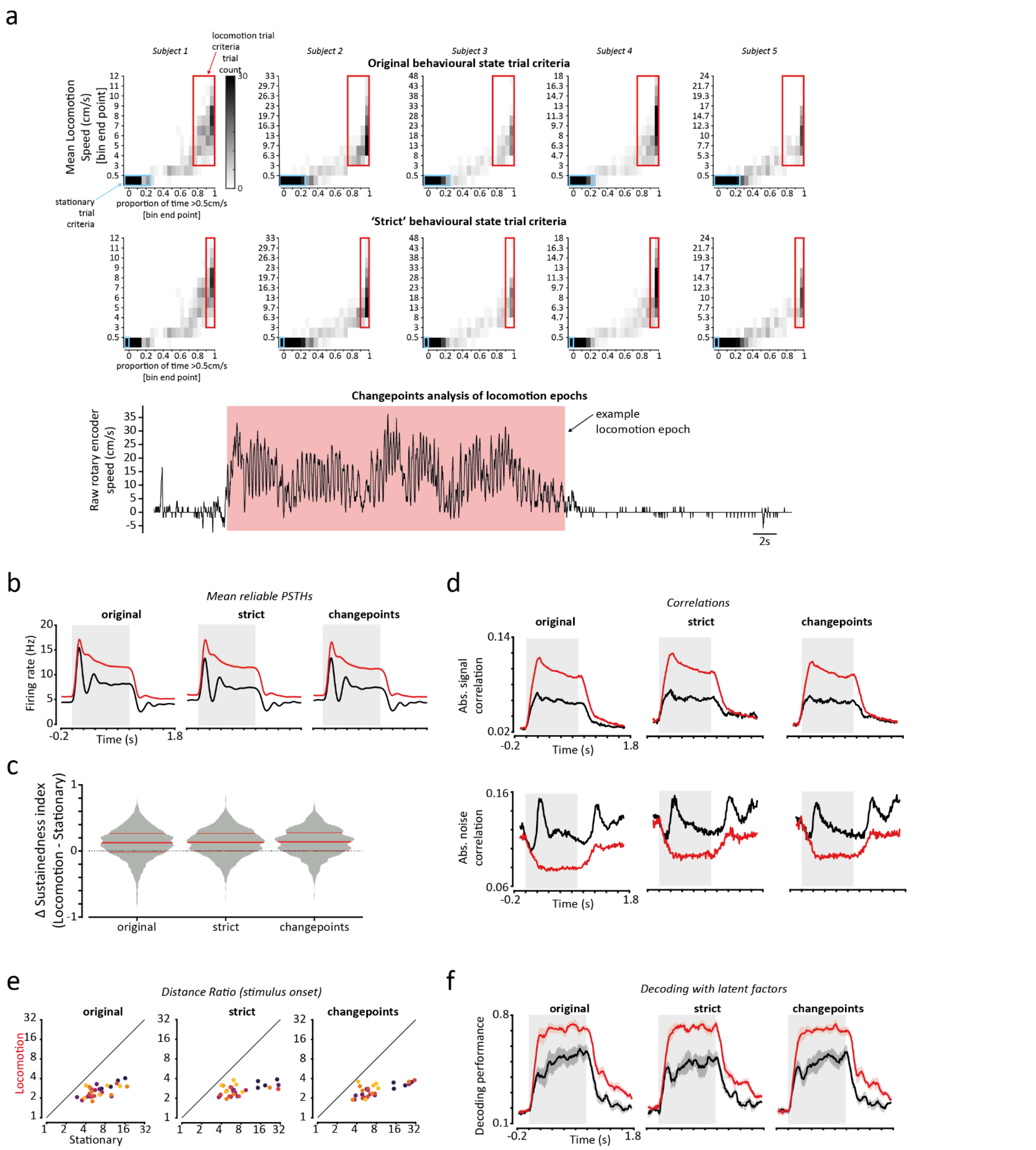

Supplementary Figure 4: Robustness of results to different behavioural state criteria
a) Overview of the three different behavioural state criteria that we compared our results across. Top row: 2D histograms of time spent > 0.5cm/s during the 2s trial periods and the mean locomotion speed during trials, for each subject. Red and blue and blue boxes indicate trials classified as locomotion and stationary respectively. Middle row: same as top row for the 'strict' criteria. Bottom row: illustration of a locomotion epoch identified using a changepoints analysis[96]. Stationary trials were classified using the same criteria as 'strict' for this classification. **b)** Comparison of mean reliable PSTH responses across the three behavioural state criteria. **c-f)** Same as b) for distributions of difference in sustainedess index between locomotion and stationary states, where the 3 horizontal red lines indicate the quartiles of each distribution (c); mean absolute signal (top row) and noise (bottom row) correlations (d); distance ratios for the stimulus onset period of responses (e); decoding performance using latent factors (f).

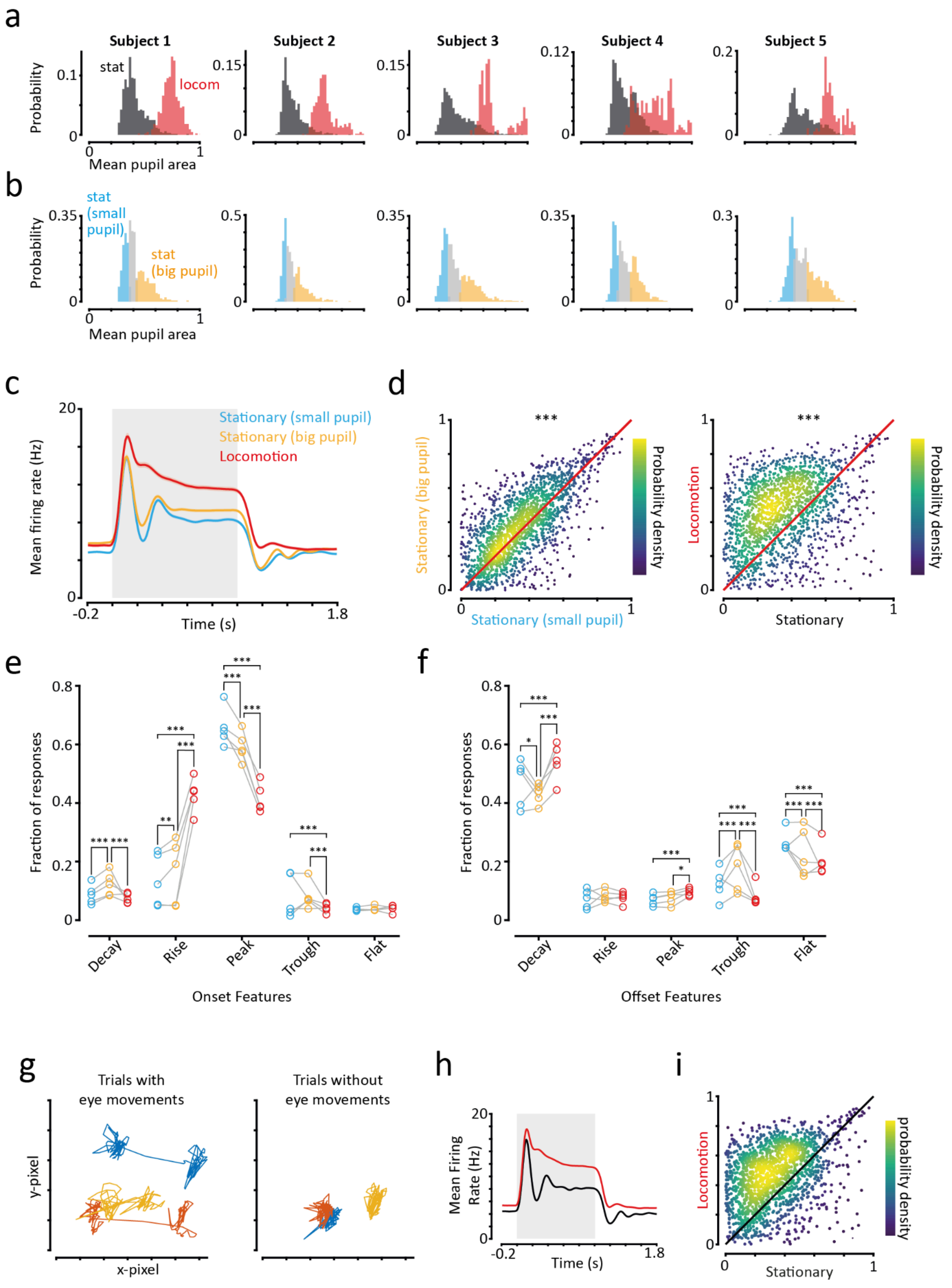

Supplementary Figure 5: Effects of pupil dilation and eye movements on single neuron responses

To determine if arousal in the absence of locomotion had an effect on single-neuron responses we used pupil dilation as a proxy measurement for arousal.

a) Probability distributions of mean pupil dilation during stimulus periods for stationary (black) and locomotion (red) trials. Pupil dilation during stationary and locomotion trials was largely non-overlapping. **b)** We partitioned stationary trials into tertiles based on mean stimulus-period pupil dilation. We compared responses from trials in the bottom (small pupil; blue) and top (big pupil; orange) tertiles. **c)** Mean reliable PSTHs from stationary (small pupil), stationary (big pupil) and locomotion trials. **d)** Scatter density plot of sustainedness index values for paired reliable responses recorded during stationary (small pupil) and stationary (big pupil) trials (left panel) and for paired reliable responses recorded during stationary and locomotion trials (right panel; same as Figure 1h - shown for reference). LME model analysis, two-sided effect of behavioural state. *** $p=7.73x10^{-16}$ for left panel and $p=5.33x10^{-103}$ for right panel. Colorbar scales are arbitrary. **e)** Proportions of reliable responses classified with different onset features. Individual data points are paired proportions of responses from each session for stationary (small pupil; blue), stationary (big pupil; orange) and locomotion (red) trials. Horizontal lines represent the fraction of all reliable responses with a given feature. GLME model analysis, two-sided effect of behavioural state. * $p<0.05$; ** $p<0.01$; *** $p<0.001$. **f)** Same as (e) for offset features. **g)** Example random subset of 3 trials (for visualisation purposes) which were classified as containing eye movements (left panel) and not containing eye movements (right panel). **h)** Mean PSTHs for stationary (black) and locomotion (red) states for trials without eye movements (related to Figure 1e). **i)** scatter density plot of sustainedndess index for paired reliable responses for trials without eye movements (related to Figure 1h). Colorbar scale is arbitrary.
Source data are provided as a Source Data file.

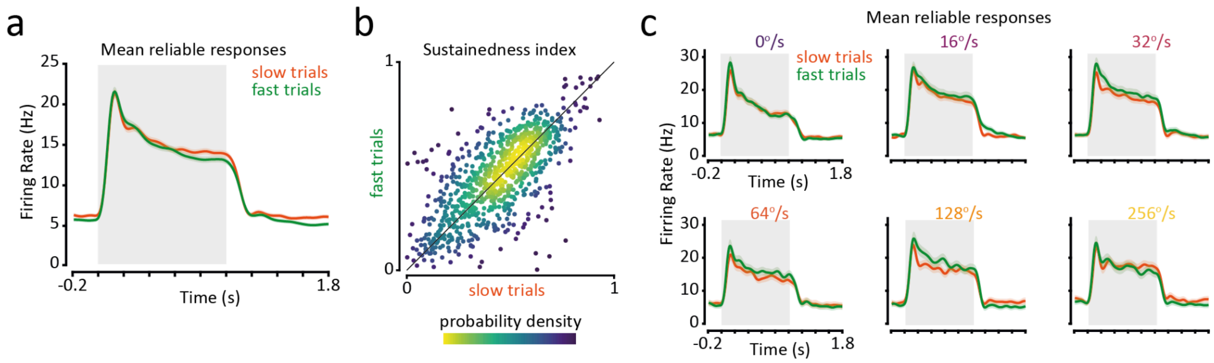

Supplementary Figure 6: Effects of locomotion speed on single neuron responses
To determine if locomotion speed had an effect on single-neuron responses we compared reliable PSTH responses computed from trials with the top ('fast') and bottom ('slow') quartiles of mean stimulus time locomotion speed for each subject. We found no evidence of systematic changes in temporal response dynamics between slow and fast locomotion speeds. However, in our experiments animals had a limited range of locomotion speeds; more extreme differences in locomotion speed may therefore be associated with changes in temporal response dynamics.
a) Mean reliable responses for 'slow' (orange) and 'fast' (green) locomotion trials. **b)** sustainedness index for paired reliable responses from slow and fast locomotion trials. Colorbar scale is arbitrary. **c)** same as (a) for each stimulus visual speed. Note that on average, there was no difference in the pattern of responses to each stimulus visual speed between slow and fast locomotion trials, reflecting the majority of single neurons.
Source data are provided as a Source Data file.

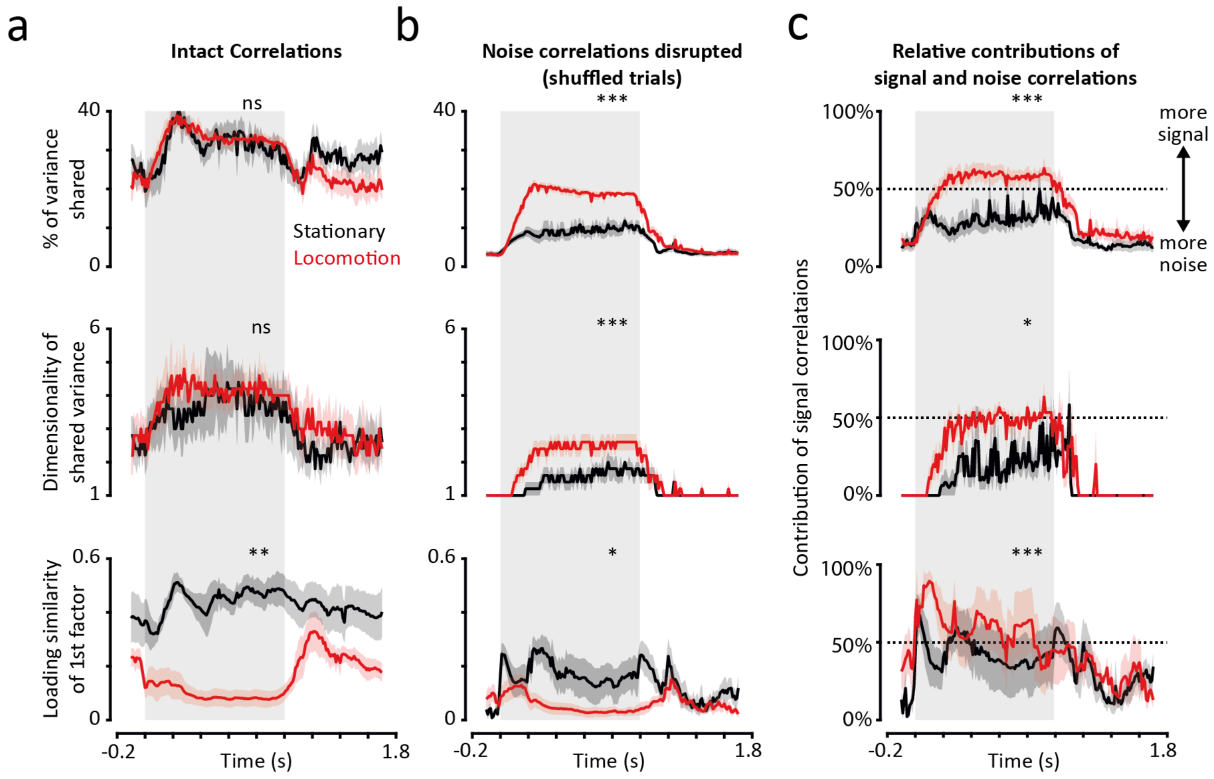

Supplementary Figure 7: Noise correlations contribute less and signal correlations contribute more to shared population activity during locomotion

We estimated the contributions of signal and noise correlations to 3 measures of shared population activity[97] by fitting independent FA models to each time window over the response period (200ms window size; 10ms step size), separately for stationary and locomotion trials. We found that disrupting noise correlations had a reduced effect on shared population activity during locomotion.

a) % of variance shared between neurons, dimensionality of shared variance and loading similarity of 1st latent factor over time, for intact population activity, separately for stationary (black) and locomotion (red) states. Significance denotes two-sided effect of behavioural state in a mixed-effects ANOVA. **b)** Same as (a) for population activity that has disrupted noise correlations. We disrupted noise correlations by shuffling trials within stimulus conditions before fitting FA models. **c)** We estimated the relative contributions of signal and noise correlations to each population measure by comparing their values for intact (a) and disrupted (b) population activity. Significance denotes interaction between behavioural state and correlation status (intact noise correlations or disrupted noise correlations) in a mixed-effects ANOVA.

ns not significant*; * p<0.05; ** p<0.01; *** p<0.001.*

Shaded regions indicate mean ± SEM across subjects (n=5).

Source data are provided as a Source Data file.

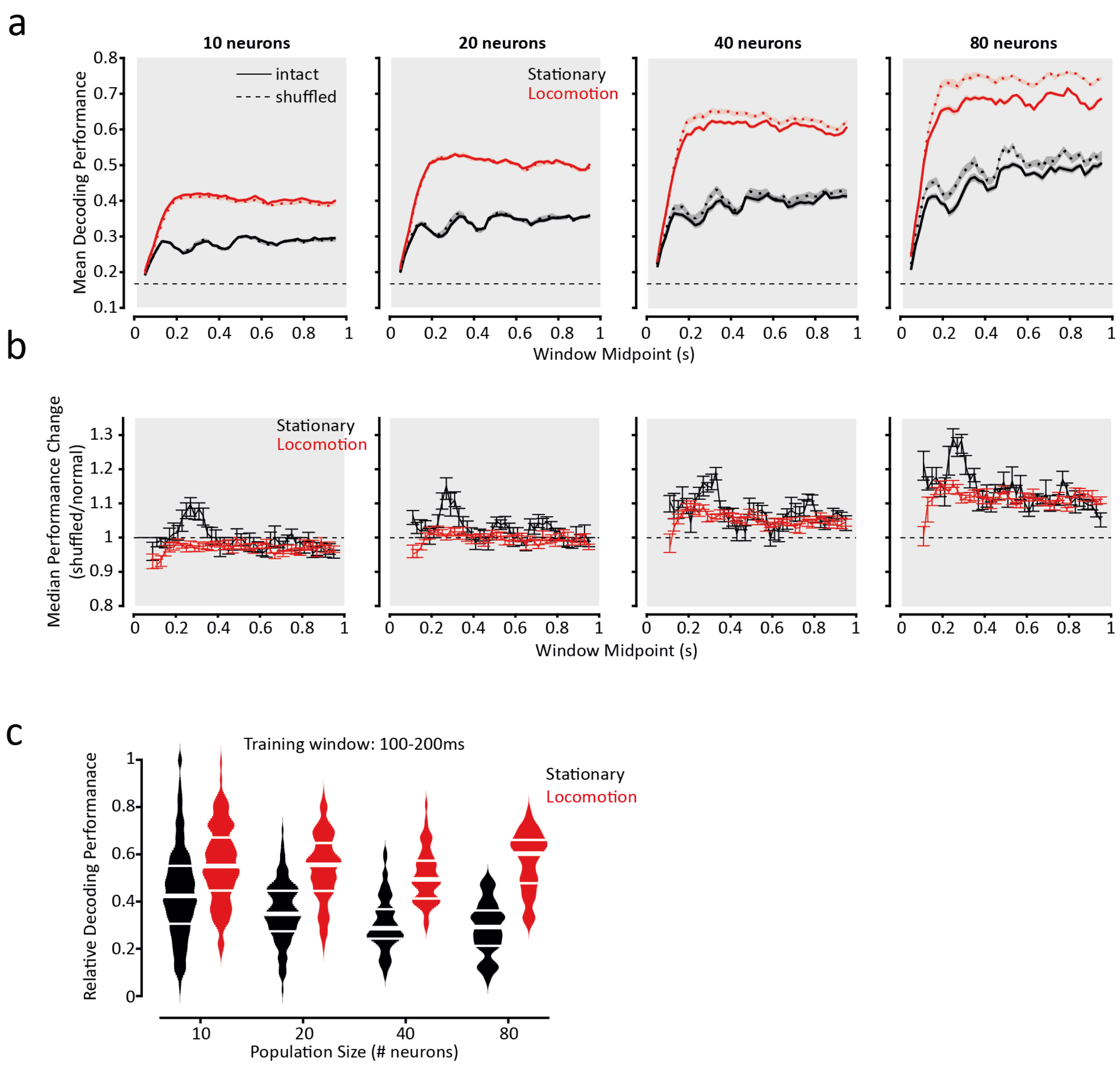

Supplementary Figure 8: Decoding analysis using different population sizes

We compared decoding performance using different population sizes.

a) Mean decoding performance using regularised LDA for 4 different population sizes (10, 20, 40 or 80 neurons), for stationary (black) and locomotion trials (red), with either intact population activity (solid lines) or trial-shuffled population activity (dashed lines) which disrupts noise correlations. Shaded regions indicate mean ± SEM across individual populations. **b)** Median Δdecoding performance between intact and shuffled population activity calculated as (shuffled performance - chance)/(intact performance - chance). Errorbars indicate ± 1 z-score of the distribution. Note the consistent increase in performance for trial-shuffled population activity for stationary trials between 0.2~0.4s following stimulus onset. **c)** Related to Figure 4e. Relative decoding performance for decoders trained in the time window 100-200ms following stimulus onset, for 4 different population sizes. Horizontal white lines indicate the median and inter-quartile range of each distribution. Note that relative decoding performance was consistent across population sizes, indicating that cross-time generalisation is higher during locomotion for this training window. Source data are provided as a Source Data file.

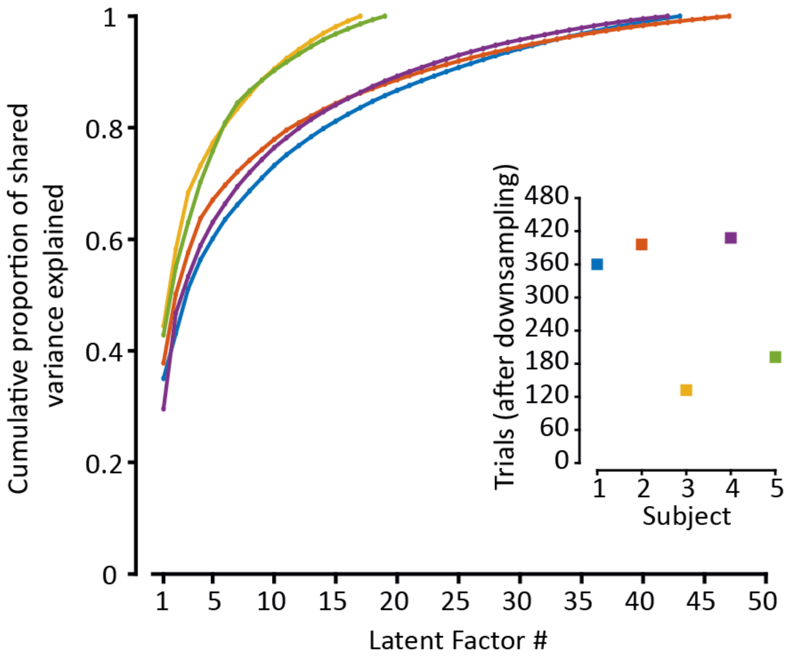

Supplementary Figure 9: Spectra of shared variance explained by latent factors
The cumulative proportion of shared variance explained is plotted as a function of the number of latent factors (in descending order of explained variance, such that factor 1 always explains the most variance) for each subject. Inset: # of trials used (after downsampling to match between conditions) to fit factor analysis models. Factor analysis models for subjects 3 and 5 had lower dimensionality due to reduced trial counts after downsampling[98].
Source data are provided as a Source Data file.

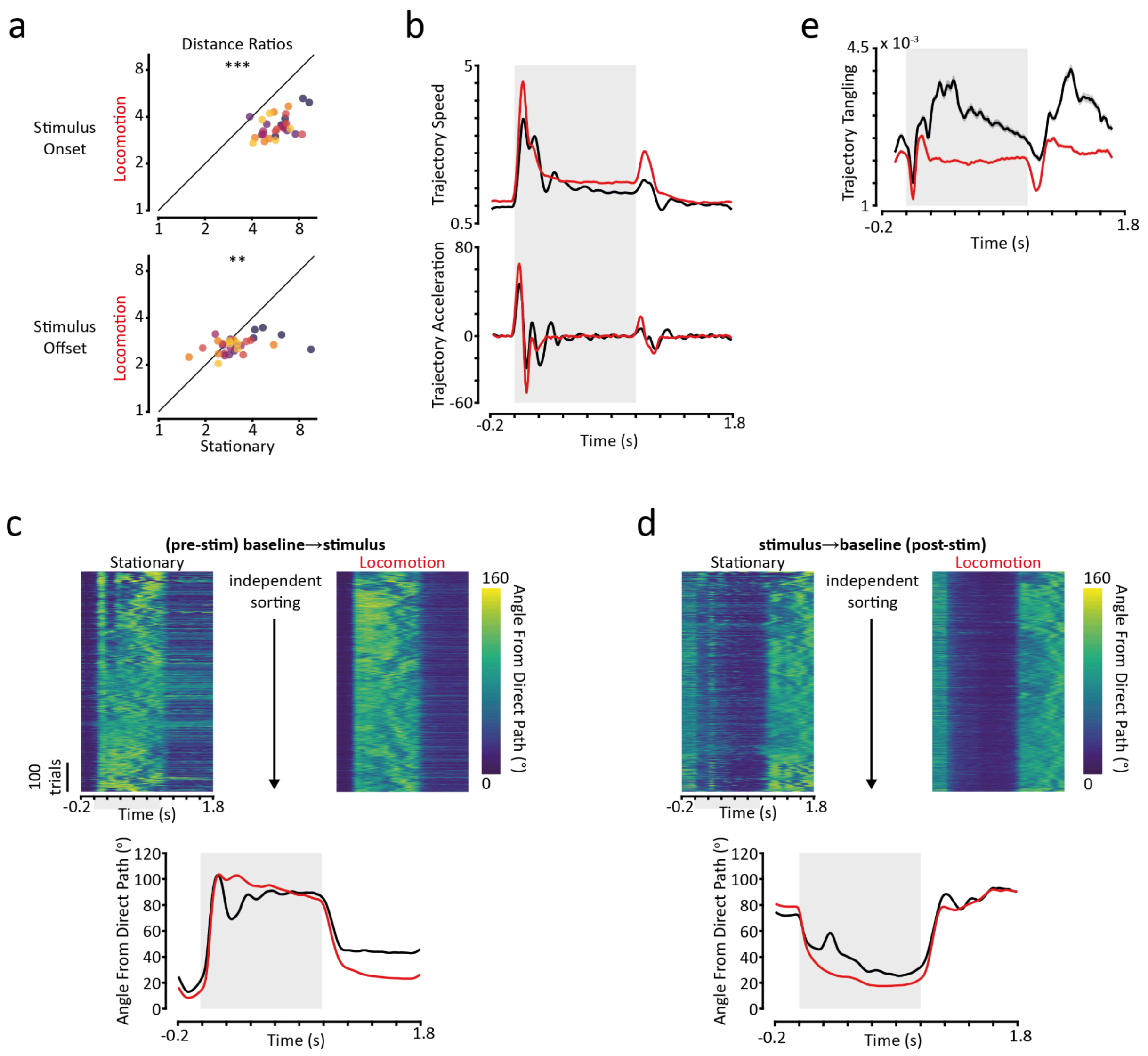

Supplementary Figure 10: Analysis of single-trial population trajectories
We performed population analyses on single-trial population trajectories and found similar results to our analysis of trial-averaged population trajectories.
a) Distance ratios for the stimulus onset and offset periods. For each stimulus condition we computed the distance ratio for each trial and then took the mean of these values. LME model analysis, effect of behavioural state. *** $p=3.76x10^{-17}$; ** $p=2.66x10^{-3}$. **b)** Mean trajectory speed (top panel) and acceleration (bottom panel) of single-trial trajectories for stationary (black) and locomotion (red) trials. **c)** Angle of approach for all trials (top panel). Stationary and locomotion trials are independently sorted. The mean angle from the direct path for stationary and locomotion trials is also shown (bottom panel). We note that averaging considerably masks the dynamics of single-trial trajectories, but that the more oscillatory dynamics in stationary trials are readily apparent in the sorted trials. The reference axis was the vector between the pre-stimulus baseline period and stimulus steady-state. **d)** Same as (c) except the reference axis was the vector between stimulus steady-state and post-stimulus period. **e)** Mean single-trial population trajectory tangling for stationary (black) and locomotion (red) trials.
Source data are provided as a Source Data file.

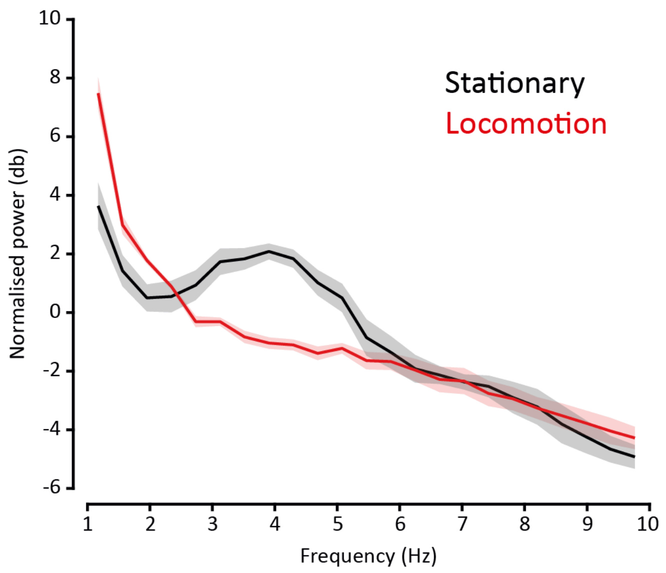

Supplementary Figure 11: 3-5Hz relative spectral power of mean population spiking activity is reduced during locomotion trials.

Normalised power spectrums of binned (non-overlapping 10ms bins) mean population activity for stationary (black) and locomotion (red) trials. Shaded regions indicate mean ± SEM across subjects (n=5).

Source data are provided as a Source Data file.

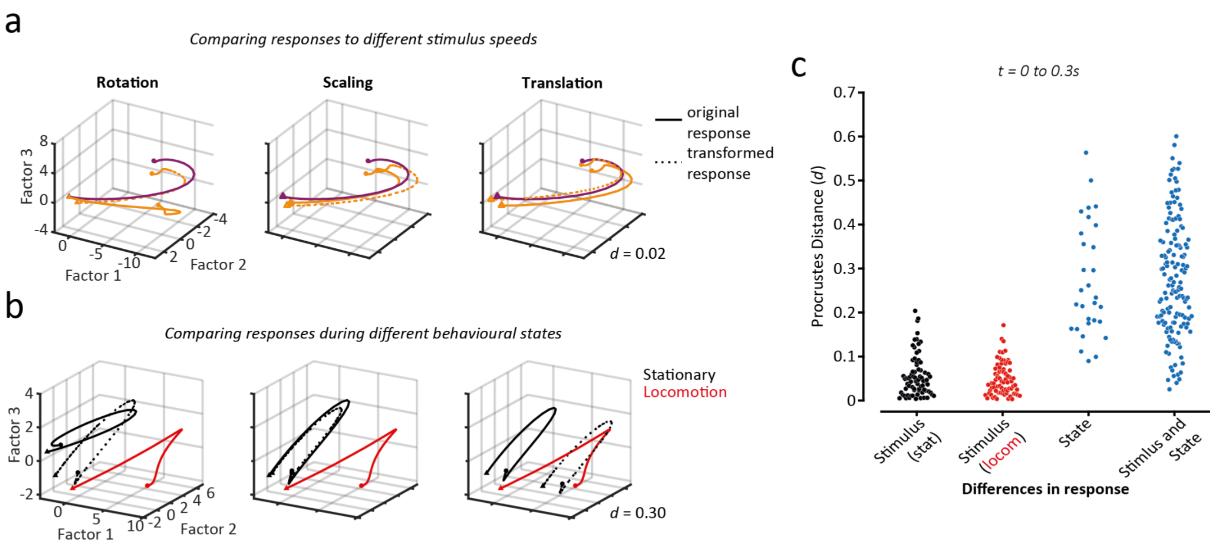

Supplementary Figure 12: Population trajectory response shapes differ more between behavioural states than stimulus speeds

We used Procrustes analysis to determine how similar different response shapes were in the period following stimulus onset (t=0 to 0.3s).

a) Example of transformation used to align population trajectory responses to 2 different stimulus speeds. The orange coloured line is the source response and the purple coloured line is the target response to align to. At each stage, the dashed orange line represents the transformed response following rotation (left panel), rotation + scaling (middle panel), and rotation + scaling + translation (right panel). The final Procrustes distance, *d*, is indicated below the rightmost panel. **b)** Same as a (a) for population trajectory responses from stationary (black) and locomotion (red) states. **c)** Procrustes distance values for pairs of population trajectory responses (t=0 to 0.3s) to different stimulus speeds ('Stimulus') and during different behavioural states ('State').

Source data are provided as a Source Data file.
