# Supplementary material for: Flexible neural population dynamics govern the speed and stability of sensory encoding in mouse visual cortex
Source: Nat Commun. 2024 Jul 30;15:6415. doi: 10.1038/s41467-024-50563-y (PMC11289260; doi:10.1038/s41467-024-50563-y)
Supplement: Supplementary file 3 — Description of additional supplementary files [file 41467_2024_50563_MOESM3_ESM.pdf]

Description of Additional Supplementary Files

Supplementary Movie 1: Example trialaveraged population trajectory. Left panel: Example trial-averaged population trajectory over the response period (Mouse 4, stimulus speed = 32°/s, behavioural state = stationary). The trajectory moves from a baseline state to a stimulus steady-state following stimulus onset and returns to the baseline state following stimulus offset. The top 3 latent factors are shown for visualisation. Right panels: Trajectory speed (top) and acceleration (bottom), over the response period. For visualisation purposes the speed and acceleration were calculated based on the 3 latent factors shown.

Supplementary Movie 2: Comparison of population trajectories from stationary and locomotion trials. Trial-averaged population trajectory responses (Mouse 2, stimulus speed = 128°/s) from stationary (black) and locomotion (red) conditions. The top 3 latent factors are shown for visualisation purposes. The trajectories exhibit distinct temporal dynamics, particularly during the stimulus onset period (t=0s to t=0.5s).

Supplementary Movie 3: Comparison of population trajectory responses to all stimulus speeds shown, for stationary and locomotion trials. Trial-averaged population trajectory responses to the six visual speeds presented (0, 16, 32, 64, 128, 256°/s; lighter colours indicate faster speeds) from stationary (left panel) and locomotion (right panel) conditions, for an example subject (Mouse 2).
