# Supplementary material for: Flexible neural population dynamics govern the speed and stability of sensory encoding in mouse visual cortex
Source: Nat Commun. 2024 Jul 30;15:6415. doi: 10.1038/s41467-024-50563-y (PMC11289260; doi:10.1038/s41467-024-50563-y)
Supplement: Supplementary file 7 — Reporting Summary [file 41467_2024_50563_MOESM7_ESM.pdf]

nature portfolio

Corresponding author(s): Edward Horrocks
Aman Saleem

Last updated by author(s): Jul 1, 2024

Reporting Summary

Nature Portfolio wishes to improve the reproducibility of the work that we publish. This form provides structure for consistency and transparency in reporting. For further information on Nature Portfolio policies, see our Editorial Policies and the Editorial Policy Checklist.

Statistics

For all statistical analyses, confirm that the following items are present in the figure legend, table legend, main text, or Methods section.

n/a	Confirmed	
☐	☒	The exact sample size (*n*) for each experimental group/condition, given as a discrete number and unit of measurement
☐	☒	A statement on whether measurements were taken from distinct samples or whether the same sample was measured repeatedly
☐	☒	The statistical test(s) used AND whether they are one- or two-sided<br>*Only common tests should be described solely by name; describe more complex techniques in the Methods section.*
☐	☒	A description of all covariates tested
☐	☒	A description of any assumptions or corrections, such as tests of normality and adjustment for multiple comparisons
☐	☒	A full description of the statistical parameters including central tendency (e.g. means) or other basic estimates (e.g. regression coefficient) AND variation (e.g. standard deviation) or associated estimates of uncertainty (e.g. confidence intervals)
☐	☒	For null hypothesis testing, the test statistic (e.g. *F*, *t*, *r*) with confidence intervals, effect sizes, degrees of freedom and *P* value noted<br>*Give P values as exact values whenever suitable.*
☒	☐	For Bayesian analysis, information on the choice of priors and Markov chain Monte Carlo settings
☒	☐	For hierarchical and complex designs, identification of the appropriate level for tests and full reporting of outcomes
☐	☒	Estimates of effect sizes (e.g. Cohen's *d*, Pearson's *r*), indicating how they were calculated

Our web collection on statistics for biologists contains articles on many of the points above.

Software and code

Policy information about availability of computer code

Data collection	Bonsai-rx 2.8 (https://bonsai-rx.org/)<br>SpikeGLX (https://billkarsh.github.io/SpikeGLX/)
Data analysis	Matlab 2022a (https://uk.mathworks.com/products/matlab.html)<br>Python 3.8 (https://www.python.org/)<br>Kilosort 3 (https://github.com/MouseLand/Kilosort)<br>ecephys_spike_sorting (https://github.com/jenniferColonell/ecephys_spike_sorting)<br>DeepLabCut (https://github.com/DeepLabCut/DeepLabCut)<br>Custom analysis code (https://github.com/eabhorrocks/Horrocksetal24_TemporalDynamics; archived at https://doi.org/10.5281/zenodo.11639115)

For manuscripts utilizing custom algorithms or software that are central to the research but not yet described in published literature, software must be made available to editors and reviewers. We strongly encourage code deposition in a community repository (e.g. GitHub). See the Nature Portfolio guidelines for submitting code & software for further information.

Data

Policy information about availability of data

All manuscripts must include a data availability statement. This statement should provide the following information, where applicable:

- Accession codes, unique identifiers, or web links for publicly available datasets
- A description of any restrictions on data availability
- For clinical datasets or third party data, please ensure that the statement adheres to our policy

Data is available on Figshare (https://doi.org/10.6084/m9.figshare.26031226.v1)
Allen Institute dataset is available from https://allensdk.readthedocs.io/en/latest/visual_coding_neuropixels.html

Research involving human participants, their data, or biological material

Policy information about studies with human participants or human data. See also policy information about sex, gender (identity/presentation), and sexual orientation and race, ethnicity and racism.

Reporting on sex and gender	n/a
Reporting on race, ethnicity, or other socially relevant groupings	n/a
Population characteristics	n/a
Recruitment	n/a
Ethics oversight	n/a

Note that full information on the approval of the study protocol must also be provided in the manuscript.

Field-specific reporting

Please select the one below that is the best fit for your research. If you are not sure, read the appropriate sections before making your selection.

☒ Life sciences ☐ Behavioural & social sciences ☐ Ecological, evolutionary & environmental sciences

For a reference copy of the document with all sections, see nature.com/documents/nr-reporting-summary-flat.pdf

Life sciences study design

All studies must disclose on these points even when the disclosure is negative.

Sample size	The number of animals (sample size) was determined based on species-specific standards for replicability in neural recordings. n = 5 mice (original data), n = 24 mice (Allen Institute Dataset).
Data exclusions	No data from the originally collected dataset were excluded from analysis. Data from the Allen Institute dataset were excluded based on insufficient trial counts (minimum of n=10 trials per condition).
Replication	Findings were consistent across mice. Relevant findings were consistent between independently collected datasets (original dataset and Allen Institute Dataset)
Randomization	The order of stimulus presentation was randomized for each session.
Blinding	Investigators were not blinded to stimulus condition during analysis, as is standard in the field.

Reporting for specific materials, systems and methods

We require information from authors about some types of materials, experimental systems and methods used in many studies. Here, indicate whether each material, system or method listed is relevant to your study. If you are not sure if a list item applies to your research, read the appropriate section before selecting a response.

Materials & experimental systems

n/a	Involved in the study
☒	☐ Antibodies
☒	☐ Eukaryotic cell lines
☒	☐ Palaeontology and archaeology
☐	☒ Animals and other organisms
☒	☐ Clinical data
☒	☐ Dual use research of concern
☒	☐ Plants

Methods

n/a	Involved in the study
☒	☐ ChIP-seq
☒	☐ Flow cytometry
☒	☐ MRI-based neuroimaging

Animals and other research organisms

Policy information about studies involving animals; ARRIVE guidelines recommended for reporting animal research, and Sex and Gender in Research

Laboratory animals	Our dataset included recordings from Mus musculus, C57BL/6J wild-type mice (n = 5; all female, age 9-18 weeks during recordings). The Allen Institute for Brain Science dataset included recordings from 24 mice (n = 8 female, n = 16 male; n = 12 wild-type C57BL/6J, n = 3 Pvalb-IRES-Cre × Ai32, n = 6 Sst-IRES-Cre × Ai32, n = 3 Vip-IRES-Cre × Ai32; age 15-20 weeks during recordings).
Wild animals	n/a
Reporting on sex	A mix of male and female mice were analysed in this study.
Field-collected samples	n/a
Ethics oversight	All experiments were performed at University College London in accordance with the Animals (Scientific Procedures) Act 1986 (United Kingdom) and Home Office (United Kingdom) approved project and personal licences.

Note that full information on the approval of the study protocol must also be provided in the manuscript.

Plants

Seed stocks	n/a
Novel plant genotypes	n/a
Authentication	n/a
