# Supplementary material for: Flexible neural population dynamics govern the speed and stability of sensory encoding in mouse visual cortex
Source: Nat Commun. 2024 Jul 30;15:6415. doi: 10.1038/s41467-024-50563-y (PMC11289260; doi:10.1038/s41467-024-50563-y)
Supplement: Supplementary file 8 — Source Data [file 41467_2024_50563_MOESM8_ESM.zip › CorrelationsStats.html]

|  | A | B | C | D | E | F | G | H | I | J | K | L | M | N | O | P | Q | R | S | T | U | V | W | X | Y | Z | AA | AB |
| --- | --- | --- | --- | --- | --- | --- | --- | --- | --- | --- | --- | --- | --- | --- | --- | --- | --- | --- | --- | --- | --- | --- | --- | --- | --- | --- | --- | --- |
| 1 | Question/comparison | Values | Statistical test and n numbers | stat results |  |  |  |  |  |  |  |  |  |  |  |  |  |  |  |  |  |  |  |  |  |  |  |  |
| 2 |  |  |  |  |  |  |  |  |  |  |  |  |  |  |  |  |  |  |  |  |  |  |  |  |  |  |  |  |
| 3 | Magnitude of correlataions | Mean (SEM) | Mean abs value over stimulus period + LME on pairs |  |  |  |  |  |  |  |  |  |  |  |  |  |  |  |  |  |  |  |  |  |  |  |  |  |
| 4 | Noise Corrs | Stat: 0.0960 (0.0007) | f = 'vals ~ state + (1|pair) + (1|sesh)'; |  |  |  |  |  |  |  |  |  |  |  |  |  |  |  |  |  |  |  |  |  |  |  |  |  |
| 5 |  | Run: 0.0607 (0.0005) | nSeshpairs = 9730 4095 2556 2485 666 | pVal = 0 |  |  |  |  |  |  |  |  |  |  |  |  |  |  |  |  |  |  |  |  |  |  |  |  |
| 6 |  |  | Total pairs = 19532 |  |  |  |  |  |  |  |  |  |  |  |  |  |  |  |  |  |  |  |  |  |  |  |  |  |
| 7 |  |  |  |  |  |  |  |  |  |  |  |  |  |  |  |  |  |  |  |  |  |  |  |  |  |  |  |  |
| 8 | Signal Corrs | Stat: 0.0440 (0.0003) |  | pVal=0 |  |  |  |  |  |  |  |  |  |  |  |  |  |  |  |  |  |  |  |  |  |  |  |  |
| 9 |  | Run: 0.0767 (0.0006) |  |  |  |  |  |  |  |  |  |  |  |  |  |  |  |  |  |  |  |  |  |  |  |  |  |  |
| 10 |  |  |  |  |  |  |  |  |  |  |  |  |  |  |  |  |  |  |  |  |  |  |  |  |  |  |  |  |
| 11 |  |  |  |  |  |  |  |  |  |  |  |  |  |  |  |  |  |  |  |  |  |  |  |  |  |  |  |  |
| 12 | Slope of signal and noise correlations |  | RM-anova, n = 5 subjects. |  |  |  |  |  |  |  |  |  |  |  |  |  |  |  |  |  |  |  |  |  |  |  |  |  |
| 13 |  |  | [p,tbl,stats,terms] = anovan(slopeVec,{timeVec,stateVec,subjVec},'model',2,'random',3,'varnames',{'Time','State','Subj'}); |  |  |  |  |  |  |  |  |  |  |  |  |  |  |  |  |  |  |  |  |  |  |  |  |  |
| 14 |  |  |  |  |  |  |  |  |  |  |  |  |  |  |  |  |  |  |  |  |  |  |  |  |  |  |  |  |
| 15 |  |  |  | Source Sum Sq. d.f. Mean Sq. F Prob>F ---------------------------------------------------------  Time 10.8998 105 0.1038 2.7 0   State 11.3975 1 11.3975 11.58 0.0272  Subj 4.7841 4 1.196 1.21 0.4276  Time\*State 10.5841 105 0.1008 2.77 0   Time\*Subj 16.1564 420 0.0385 1.06 0.2907  State\*Subj 3.9355 4 0.9839 26.99 0   Error 15.3096 420 0.0365   Total 73.067 1059 |  |  |  |  |  |  |  |  |  |  |  |  |  |  |  |  |  |  |  |  |  |  |  |  |
| 16 |  |  |  |  |  |  |  |  |  |  |  |  |  |  |  |  |  |  |  |  |  |  |  |  |  |  |  |  |
| 17 | Stabilisation of signal and noise |  | RM ANOVAs on non-overlapping time windows, from t=0 to t=800. n= 5 subjects. |  |  |  |  |  |  |  |  |  |  |  |  |  |  |  |  |  |  |  |  |  |  |  |  |  |
| 18 |  |  | [p,tbl,stats,terms] = anovan(noiseVec,{timeVec,stateVec,subjVec},'model',2,'random',3,'varnames',{'Time','State','Subj'}); |  |  |  |  |  |  |  |  |  |  |  |  |  |  |  |  |  |  |  |  |  |  |  |  |  |
| 19 |  |  |  |  |  |  |  |  |  |  |  |  |  |  |  |  |  |  |  |  |  |  |  |  |  |  |  |  |
| 20 |  |  | Noise corrs | ---------------------------------------------------------  Source Sum Sq. d.f. Mean Sq. F Prob>F ---------------------------------------------------------  Time 4.0321 80 0.0504 19.9 0   State 1.2595 1 1.25953 9.04 0.0397  Subj 4.7597 4 1.18993 8.47 0.0301  Time\*State 0.5948 80 0.00744 5.12 0   Time\*Subj 0.8106 320 0.00253 1.75 0   State\*Subj 0.5575 4 0.13937 96.01 0   Error 0.4645 320 0.00145   Total 12.4788 809 |  |  |  |  |  |  |  |  |  |  |  |  |  |  |  |  |  |  |  |  |  |  |  |  |
| 21 |  |  |  |  |  |  |  |  |  |  |  |  |  |  |  |  |  |  |  |  |  |  |  |  |  |  |  |  |
| 22 |  |  | Signal Corrs | ---------------------------------------------------------   Source Sum Sq. d.f. Mean Sq. F Prob>F ---------------------------------------------------------  Time 53.9354 80 0.67419 81.63 0   State 6.3209 1 6.32093 21.69 0.0096  Subj 3.6868 4 0.9217 3.11 0.1445  Time\*State 1.4626 80 0.01828 6.12 0   Time\*Subj 2.643 320 0.00826 2.76 0   State\*Subj 1.1659 4 0.29147 97.52 0   Error 0.9565 320 0.00299   Total 70.1711 809 |  |  |  |  |  |  |  |  |  |  |  |  |  |  |  |  |  |  |  |  |  |  |  |  |
| 23 |  |  |  |  |  |  |  |  |  |  |  |  |  |  |  |  |  |  |  |  |  |  |  |  |  |  |  |  |
| 24 |  |  |  |  |  |  |  |  |  |  |  |  |  |  |  |  |  |  |  |  |  |  |  |  |  |  |  |  |
| 25 |  |  | Test on traianing window (100-200) |  |  |  |  |  |  |  |  |  |  |  |  |  |  |  |  |  |  |  |  |  |  |  |  |  |
| 26 |  |  | n=5 subjects | AD-test for normaility of difference, p = 0.1614 |  |  |  |  |  |  |  |  |  |  |  |  |  |  |  |  |  |  |  |  |  |  |  |  |
| 27 |  |  |  | paird-tt test, p = 0.0137 |  |  |  |  |  |  |  |  |  |  |  |  |  |  |  |  |  |  |  |  |  |  |  |  |
| 28 |  |  |  |  |  |  |  |  |  |  |  |  |  |  |  |  |  |  |  |  |  |  |  |  |  |  |  |  |
| 29 |  |  |  |  |  |  |  |  |  |  |  |  |  |  |  |  |  |  |  |  |  |  |  |  |  |  |  |  |
| 30 | Shared population activity |  |  |  |  |  |  |  |  |  |  |  |  |  |  |  |  |  |  |  |  |  |  |  |  |  |  |  |
| 31 | Shared Variance | RM-ANOVA, 5 subjs | [p,tbl,stats,terms] = anovan(vals,{timeVec,stateVec,subjVec},'model',2,'random',3,'varnames',{'Time','State','Subj'}); |  |  |  |  |  |  |  |  |  |  |  |  |  |  |  |  |  |  |  |  |  |  |  |  |  |
| 32 |  |  | Intact noise correlations: | ---------------------------------------------------------  Source Sum Sq. d.f. Mean Sq. F Prob>F ----------------------------------------------------------  Time 38797.1 180 215.5 13.59 0   State 725.7 1 725.7 0.7 0.4508  Subj 42673.9 4 10668.5 10.24 0.0223  Time\*State 7469.4 180 41.5 2.66 0   Time\*Subj 11421.1 720 15.9 1.02 0.4109  State\*Subj 4165.4 4 1041.4 66.76 0   Error 11230.8 720 15.6   Total 116483.5 1809 |  |  |  |  |  |  |  |  |  |  |  |  |  |  |  |  |  |  |  |  |  |  |  |  |
| 33 |  |  |  |  |  |  |  |  |  |  |  |  |  |  |  |  |  |  |  |  |  |  |  |  |  |  |  |  |
| 34 |  |  | Disrupted noise corelations (shuffled) | ---------------------------------------------------------    Source Sum Sq. d.f. Mean Sq. F Prob>F  ---------------------------------------------------------  Time 42871.8 180 238.2 39.44 0   State 12404.8 1 12404.8 78.84 0.0009  Subj 2032.7 4 508.2 3.17 0.1402  Time\*State 9714.1 180 54 18.83 0   Time\*Subj 4347.6 720 6 2.11 0   State\*Subj 629.4 4 157.4 54.91 0   Error 2063.3 720 2.9   Total 74063.8 1809 |  |  |  |  |  |  |  |  |  |  |  |  |  |  |  |  |  |  |  |  |  |  |  |  |
| 35 |  |  |  |  |  |  |  |  |  |  |  |  |  |  |  |  |  |  |  |  |  |  |  |  |  |  |  |  |
| 36 |  |  |  |  |  |  |  |  |  |  |  |  |  |  |  |  |  |  |  |  |  |  |  |  |  |  |  |  |
| 37 |  |  | Mixed model with shuffled corrs as a factor | ---------------------------------------------------------    Source Sum Sq. d.f. Mean Sq. F Prob>F  -----------------------------------------------------------  Time 76768.3 180 426.5 34.21 0   State 3564.8 1 3564.8 4.18 0.1103  Subj 16416.6 4 4104.1 0.52 0.728   Corr 344715.3 1 344715.3 48.74 0.0022  Time\*State 13960.4 180 77.6 7.36 0   Time\*Subj 8976.8 720 12.5 1.18 0.0022  Time\*Corr 4900.7 180 27.2 2.58 0   State\*Subj 3409 4 852.3 80.9 0   State\*Corr 9565.7 1 9565.7 907.97 0   Subj\*Corr 28290.1 4 7072.5 671.31 0   Error 24694.9 2344 10.5   Total 535262.6 3619 |  |  |  |  |  |  |  |  |  |  |  |  |  |  |  |  |  |  |  |  |  |  |  |  |
| 38 |  |  | [p,tbl,stats,terms] = anovan(vals,{timeVec2,stateVec2,subjVec2, corrVec},'model','interaction','random',3,'varnames',{'Time','State','Subj','Corr'}); |  |  |  |  |  |  |  |  |  |  |  |  |  |  |  |  |  |  |  |  |  |  |  |  |  |
| 39 |  |  |  |  |  |  |  |  |  |  |  |  |  |  |  |  |  |  |  |  |  |  |  |  |  |  |  |  |
| 40 | Dimensionality of Shared Variance |  | Intact | ---------------------------------------------------------    Source Sum Sq. d.f. Mean Sq. F Prob>F  ---------------------------------------------------------  Time 778.92 180 4.327 6.61 0   State 60.17 1 60.166 3.33 0.142   Subj 2322.85 4 580.712 32.13 0.0027  Time\*State 97.03 180 0.539 0.85 0.9142  Time\*Subj 471.55 720 0.655 1.03 0.3542  State\*Subj 72.22 4 18.055 28.35 0   Error 458.58 720 0.637   Total 4261.32 1809 |  |  |  |  |  |  |  |  |  |  |  |  |  |  |  |  |  |  |  |  |  |  |  |  |
| 41 |  |  |  |  |  |  |  |  |  |  |  |  |  |  |  |  |  |  |  |  |  |  |  |  |  |  |  |  |
| 42 |  |  | Shuffled | ---------------------------------------------------------    Source Sum Sq. d.f. Mean Sq. F Prob>F  ---------------------------------------------------------  Time 433.263 180 2.407 13.59 0   State 95.611 1 95.611 94.9 0.0006  Subj 80.306 4 20.0765 18.05 0.0039  Time\*State 88.189 180 0.4899 6.76 0   Time\*Subj 127.494 720 0.1771 2.44 0   State\*Subj 4.03 4 1.0075 13.9 0   Error 52.17 720 0.0725   Total 881.063 1809 |  |  |  |  |  |  |  |  |  |  |  |  |  |  |  |  |  |  |  |  |  |  |  |  |
| 43 |  |  |  |  |  |  |  |  |  |  |  |  |  |  |  |  |  |  |  |  |  |  |  |  |  |  |  |  |
| 44 |  |  | Mixed-model | ---------------------------------------------------------    Source Sum Sq. d.f. Mean Sq. F Prob>F  ----------------------------------------------------------  Time 1124.19 180 6.245 13.17 0   State 153.73 1 153.734 16.32 0.0156  Subj 1591.73 4 397.932 1.88 0.2681  Corr 3001 1 3000.999 14.79 0.0184  Time\*State 107.67 180 0.598 1.59 0   Time\*Subj 341.47 720 0.474 1.26 0.0001  Time\*Corr 88 180 0.489 1.3 0.0064  State\*Subj 37.69 4 9.422 24.97 0   State\*Corr 2.04 1 2.043 5.41 0.0201  Subj\*Corr 811.43 4 202.857 537.62 0   Error 884.44 2344 0.377   Total 8143.39 3619 |  |  |  |  |  |  |  |  |  |  |  |  |  |  |  |  |  |  |  |  |  |  |  |  |
| 45 |  |  |  |  |  |  |  |  |  |  |  |  |  |  |  |  |  |  |  |  |  |  |  |  |  |  |  |  |
| 46 |  |  |  |  |  |  |  |  |  |  |  |  |  |  |  |  |  |  |  |  |  |  |  |  |  |  |  |  |
| 47 | Loading similarity of first dimension |  | Intact | ---------------------------------------------------------    Source Sum Sq. d.f. Mean Sq. F Prob>F  ----------------------------------------------------------  Time 2.2676 180 0.0126 1.29 0.0133  State 34.1743 1 34.1743 60.47 0.0015  Subj 9.1068 4 2.2767 3.99 0.102   Time\*State 4.5616 180 0.0253 6.89 0   Time\*Subj 7.048 720 0.0098 2.66 0   State\*Subj 2.2604 4 0.5651 153.6 0   Error 2.649 720 0.0037   Total 62.0677 1809 |  |  |  |  |  |  |  |  |  |  |  |  |  |  |  |  |  |  |  |  |  |  |  |  |
| 48 |  |  |  |  |  |  |  |  |  |  |  |  |  |  |  |  |  |  |  |  |  |  |  |  |  |  |  |  |
| 49 |  |  | Shuffled | ---------------------------------------------------------    Source Sum Sq. d.f. Mean Sq. F Prob>F  ---------------------------------------------------------  Time 2.1291 180 0.01183 1.33 0.006   State 3.1972 1 3.19722 12.38 0.0245  Subj 1.0949 4 0.27374 1.05 0.4812  Time\*State 2.0078 180 0.01115 2.05 0   Time\*Subj 6.4 720 0.00889 1.64 0   State\*Subj 1.0329 4 0.25822 47.51 0   Error 3.9132 720 0.00543   Total 19.7751 1809 |  |  |  |  |  |  |  |  |  |  |  |  |  |  |  |  |  |  |  |  |  |  |  |  |
| 50 |  |  |  |  |  |  |  |  |  |  |  |  |  |  |  |  |  |  |  |  |  |  |  |  |  |  |  |  |
| 51 |  |  | Mixed-model | ---------------------------------------------------------    Source Sum Sq. d.f. Mean Sq. F Prob>F  -----------------------------------------------------------  Time 2.024 180 0.0112 0.75 0.9904  State 29.139 1 29.1386 134.83 0.0003  Subj 2.367 4 0.5919 0.27 0.8844  Corr 32.74 1 32.7396 16.72 0.015   Time\*State 5.573 180 0.031 5.74 0   Time\*Subj 10.79 720 0.015 2.78 0   Time\*Corr 2.372 180 0.0132 2.44 0   State\*Subj 0.864 4 0.2161 40.06 0   State\*Corr 8.233 1 8.2329 1526.12 0   Subj\*Corr 7.834 4 1.9586 363.06 0   Error 12.645 2344 0.0054   Total 114.582 3619 |  |  |  |  |  |  |  |  |  |  |  |  |  |  |  |  |  |  |  |  |  |  |  |  |
