# Supplementary material for: Flexible neural population dynamics govern the speed and stability of sensory encoding in mouse visual cortex
Source: Nat Commun. 2024 Jul 30;15:6415. doi: 10.1038/s41467-024-50563-y (PMC11289260; doi:10.1038/s41467-024-50563-y)
Supplement: Supplementary file 8 — Source Data [file 41467_2024_50563_MOESM8_ESM.zip › SingleNeuronRespsStats_And_S1_S2_S3_S5.html]

|  | A | B | C | D | E | F | G | H | I | J | K | L | M | N | O | P | Q | R | S | T | U | V | W | X | Y | Z |
| --- | --- | --- | --- | --- | --- | --- | --- | --- | --- | --- | --- | --- | --- | --- | --- | --- | --- | --- | --- | --- | --- | --- | --- | --- | --- | --- |
| 1 | Question/comparison | Values | Statistical test and n numbers | stat results |  |  |  |  |  |  |  |  |  |  |  |  |  |  |  |  |  |  |  |  |  |  |
| 2 |  |  |  |  |  |  |  |  |  |  |  |  |  |  |  |  |  |  |  |  |  |  |  |  |  |  |
| 3 | All responses |  |  |  |  |  |  |  |  |  |  |  |  |  |  |  |  |  |  |  |  |  |  |  |  |  |
| 4 | # reliable responses | stat/run/both | McNemar test |  |  |  |  |  |  |  |  |  |  |  |  |  |  |  |  |  |  |  |  |  |  |  |
| 5 | does proportion of reliable responses vary between states? | Total responses = 9,498 (per state) | (+stat+run, +stat-run -stat+run, -stat-run) | p=0 |  |  |  |  |  |  |  |  |  |  |  |  |  |  |  |  |  |  |  |  |  |  |
| 6 |  | Stat reliable resps = 2,077 (21.87%) | 1807 270  995 6426 |  |  |  |  |  |  |  |  |  |  |  |  |  |  |  |  |  |  |  |  |  |  |  |
| 7 |  | Run reliable resps = 2,802 (29.5%) |  |  |  |  |  |  |  |  |  |  |  |  |  |  |  |  |  |  |  |  |  |  |  |  |
| 8 |  | Both reliable resps = 1,807 (19.03%) |  |  |  |  |  |  |  |  |  |  |  |  |  |  |  |  |  |  |  |  |  |  |  |  |
| 9 |  |  |  |  |  |  |  |  |  |  |  |  |  |  |  |  |  |  |  |  |  |  |  |  |  |  |
| 10 |  |  |  |  |  |  |  |  |  |  |  |  |  |  |  |  |  |  |  |  |  |  |  |  |  |  |
| 11 | onset/offset features | Proportions (Decay, Rise, Peak, Trough, Flat) | Binomial GLMEs |  |  |  |  |  |  |  |  |  |  |  |  |  |  |  |  |  |  |  |  |  |  |  |
| 12 | does proportion of features vary between states? | propStatOn = 0.0896 0.1247 0.6856 0.0515 0.0486 | independent GLME for each feature, equation: | pVal\_onset = 0.1291    0.0000   0.0000    0.1222    0.0986 |  |  |  |  |  |  |  |  |  |  |  |  |  |  |  |  |  |  |  |  |  |  |
| 13 |  | propRunOn = 0.0774 0.4336 0.4079 0.0421 0.0389 | fet ~ state + (1|session) | pVal\_offset = 0.0000 0.0037 0.0996 0.0000 0.0000 |  |  |  |  |  |  |  |  |  |  |  |  |  |  |  |  |  |  |  |  |  |  |
| 14 |  | propStatOff =0.4612 0.0558 0.0823 0.1223 0.2783 | n numbers are stat/run reliable responses (see above) |  |  |  |  |  |  |  |  |  |  |  |  |  |  |  |  |  |  |  |  |  |  |  |
| 15 |  | propRunOff =0.5496 0.0771 0.0960 0.0846 0.1927 |  |  |  |  |  |  |  |  |  |  |  |  |  |  |  |  |  |  |  |  |  |  |  |  |
| 16 |  |  |  |  |  |  |  |  |  |  |  |  |  |  |  |  |  |  |  |  |  |  |  |  |  |  |
| 17 | sustainedness |  | LME | pValue for state = 5.3278e-103 |  |  |  |  |  |  |  |  |  |  |  |  |  |  |  |  |  |  |  |  |  |  |
| 18 | does sustainedness of responses vary between states? | Quantiles (median and IQR) | n numbers are both reliable responses |  |  |  |  |  |  |  |  |  |  |  |  |  |  |  |  |  |  |  |  |  |  |  |
| 19 |  | Stat: 0.1829 0.3193 0.4846 | sustainedness ~ state + (1|unit) + (1|session) |  |  |  |  |  |  |  |  |  |  |  |  |  |  |  |  |  |  |  |  |  |  |  |
| 20 |  | Run: 0.3229 0.4826 0.6161 |  |  |  |  |  |  |  |  |  |  |  |  |  |  |  |  |  |  |  |  |  |  |  |  |
| 21 |  |  |  |  |  |  |  |  |  |  |  |  |  |  |  |  |  |  |  |  |  |  |  |  |  |  |
| 22 | Mean and peak firing rate changes |  |  |  |  |  |  |  |  |  |  |  |  |  |  |  |  |  |  |  |  |  |  |  |  |  |
| 23 | Do mean and peak firing rates change by the same fractional amount? | Quantiles (median and IQR) | signrank(log2(meanFR\_fracChange), log2(peakFR\_fracChange)) | p = 1.5994e-138 |  |  |  |  |  |  |  |  |  |  |  |  |  |  |  |  |  |  |  |  |  |  |
| 24 |  | Mean rate (baseline corrected) | n numbers are both reliable responses |  |  |  |  |  |  |  |  |  |  |  |  |  |  |  |  |  |  |  |  |  |  |  |
| 25 |  | Stat: 1.4219 3.2061 6.2320 |  |  |  |  |  |  |  |  |  |  |  |  |  |  |  |  |  |  |  |  |  |  |  |  |
| 26 |  | Run: 1.2405 5.4136 12.1279 |  |  |  |  |  |  |  |  |  |  |  |  |  |  |  |  |  |  |  |  |  |  |  |  |
| 27 |  |  |  |  |  |  |  |  |  |  |  |  |  |  |  |  |  |  |  |  |  |  |  |  |  |  |
| 28 |  | Peak rate (baseline corrected) |  |  |  |  |  |  |  |  |  |  |  |  |  |  |  |  |  |  |  |  |  |  |  |  |
| 29 |  | Stat: 5.5777 10.0790 19.2957 |  |  |  |  |  |  |  |  |  |  |  |  |  |  |  |  |  |  |  |  |  |  |  |  |
| 30 |  | Run: 5.3432 12.9791 25.7037 |  |  |  |  |  |  |  |  |  |  |  |  |  |  |  |  |  |  |  |  |  |  |  |  |
| 31 |  |  |  |  |  |  |  |  |  |  |  |  |  |  |  |  |  |  |  |  |  |  |  |  |  |  |
| 32 |  | Fractional change in mean: 1.0999 2.0281 4.0721 |  |  |  |  |  |  |  |  |  |  |  |  |  |  |  |  |  |  |  |  |  |  |  |  |
| 33 |  | Fractional change in peak: 0.7297 1.1714 1.7186 |  |  |  |  |  |  |  |  |  |  |  |  |  |  |  |  |  |  |  |  |  |  |  |  |
| 34 |  |  |  |  |  |  |  |  |  |  |  |  |  |  |  |  |  |  |  |  |  |  |  |  |  |  |
| 35 | Visual speeds (Fig S1) |  |  |  |  |  |  |  |  |  |  |  |  |  |  |  |  |  |  |  |  |  |  |  |  |  |
| 36 | Response features | Proportions (Decay, Rise, Peak, Trough, Flat) | Binomial GLMEs as above, separate for each speed |  |  |  |  |  |  |  |  |  |  |  |  |  |  |  |  |  |  |  |  |  |  |  |
| 37 | 0 | statProp\_onset: [0.0994 0.0426 0.7983 0.0284 0.0312] | # reliable responses | pVal\_onset: [0.0064 8.1154e-17 9.5748e-13 0.0490 0.1644] |  |  |  |  |  |  |  |  |  |  |  |  |  |  |  |  |  |  |  |  |  |  |
| 38 |  | runProp\_onset: [0.0495 0.3237 0.5526 0.0577 0.0165] | nStat: 352 | pVal\_offset: [2.8582e-13 0.5581 0.0158 0.0633 1.1693e-19] |  |  |  |  |  |  |  |  |  |  |  |  |  |  |  |  |  |  |  |  |  |  |
| 39 |  | statProp\_offset: [0.2188 0.0540 0.0852 0.0284 0.6136] | nRun:485 |  |  |  |  |  |  |  |  |  |  |  |  |  |  |  |  |  |  |  |  |  |  |  |
| 40 |  | runProp\_offset: [0.4660 0.0454 0.1402 0.0557 0.2928] |  |  |  |  |  |  |  |  |  |  |  |  |  |  |  |  |  |  |  |  |  |  |  |  |
| 41 |  |  |  |  |  |  |  |  |  |  |  |  |  |  |  |  |  |  |  |  |  |  |  |  |  |  |
| 42 | 16 | statProp\_onset: [0.0628 0.2120 0.6283 0.0524 0.0445] | nStat:382 | pVal\_onset: [0.7352 3.8472e-13 2.5451e-11 0.1012 0.6849] |  |  |  |  |  |  |  |  |  |  |  |  |  |  |  |  |  |  |  |  |  |  |
| 43 |  | runProp\_onset: [0.0685 0.4476 0.4032 0.0302 0.0504] | nRun: 496 | pVal\_offset: [0.2535 0.7394 0.2914 3.2750e-04 0.7611] |  |  |  |  |  |  |  |  |  |  |  |  |  |  |  |  |  |  |  |  |  |  |
| 44 |  | statProp\_offset: [0.4791 0.0707 0.0890 0.1309 0.2304] |  |  |  |  |  |  |  |  |  |  |  |  |  |  |  |  |  |  |  |  |  |  |  |  |
| 45 |  | runProp\_offset: [0.5101 0.0766 0.1109 0.0605 0.2419] |  |  |  |  |  |  |  |  |  |  |  |  |  |  |  |  |  |  |  |  |  |  |  |  |
| 46 |  |  |  |  |  |  |  |  |  |  |  |  |  |  |  |  |  |  |  |  |  |  |  |  |  |  |
| 47 | 32 | statProp\_onset: [0.0765 0.1831 0.6311 0.0710 0.0383] | nStat:366 | pVal\_onset: [0.6839 3.4120e-20 3.2140e-17 0.0584 0.8352] |  |  |  |  |  |  |  |  |  |  |  |  |  |  |  |  |  |  |  |  |  |  |
| 48 |  | runProp\_onset: [0.0842 0.4928 0.3409 0.0411 0.0411] | nRun: 487 | pVal\_offset: [0.0057 0.4587 0.6270 0.2657 0.0052] |  |  |  |  |  |  |  |  |  |  |  |  |  |  |  |  |  |  |  |  |  |  |
| 49 |  | statProp\_offset: [0.5000 0.0628 0.0984 0.1148 0.2240] |  |  |  |  |  |  |  |  |  |  |  |  |  |  |  |  |  |  |  |  |  |  |  |  |
| 50 |  | runProp\_offset: [0.5893 0.0760 0.0883 0.0924 0.1540] |  |  |  |  |  |  |  |  |  |  |  |  |  |  |  |  |  |  |  |  |  |  |  |  |
| 51 |  |  |  |  |  |  |  |  |  |  |  |  |  |  |  |  |  |  |  |  |  |  |  |  |  |  |
| 52 | 64 | statProp\_onset: [0.0978 0.1201 0.6704 0.0447 0.0670] | nStat:358 | pVal\_onset: [0.2743 4.1914e-23 9.8567e-18 0.3337 0.5595] |  |  |  |  |  |  |  |  |  |  |  |  |  |  |  |  |  |  |  |  |  |  |
| 53 |  | runProp\_onset: [0.0763 0.4746 0.3602 0.0318 0.0572] | nRun: 472 | pVal\_offset: [0.3132 0.0016 0.4402 0.0425 0.2519] |  |  |  |  |  |  |  |  |  |  |  |  |  |  |  |  |  |  |  |  |  |  |
| 54 |  | statProp\_offset: [0.5251 0.0335 0.0866 0.1564 0.1983] |  |  |  |  |  |  |  |  |  |  |  |  |  |  |  |  |  |  |  |  |  |  |  |  |
| 55 |  | runProp\_offset: [0.5593 0.0911 0.0720 0.1102 0.1674] |  |  |  |  |  |  |  |  |  |  |  |  |  |  |  |  |  |  |  |  |  |  |  |  |
| 56 |  |  |  |  |  |  |  |  |  |  |  |  |  |  |  |  |  |  |  |  |  |  |  |  |  |  |
| 57 | 128 | statProp\_onset: [0.1061 0.0772 0.6913 0.0450 0.0804] | nStat:311 | pVal\_onset: [0.7309 5.6576e-21 3.4326e-16 0.8140 0.0176] |  |  |  |  |  |  |  |  |  |  |  |  |  |  |  |  |  |  |  |  |  |  |
| 58 |  | runProp\_onset: [0.0985 0.4420 0.3786 0.0416 0.0394] | nRun: 457 | pVal\_offset: [0.2423 0.0397 0.8032 0.0577 0.1400] |  |  |  |  |  |  |  |  |  |  |  |  |  |  |  |  |  |  |  |  |  |  |
| 59 |  | statProp\_offset: [0.5305 0.0514 0.0675 0.1640 0.1865] |  |  |  |  |  |  |  |  |  |  |  |  |  |  |  |  |  |  |  |  |  |  |  |  |
| 60 |  | runProp\_offset: [0.5733 0.0919 0.0722 0.1160 0.1466] |  |  |  |  |  |  |  |  |  |  |  |  |  |  |  |  |  |  |  |  |  |  |  |  |
| 61 |  |  |  |  |  |  |  |  |  |  |  |  |  |  |  |  |  |  |  |  |  |  |  |  |  |  |
| 62 | 256 | statProp\_onset: [0.1006 0.0942 0.7045 0.0682 0.0325] | nStat:308 | pVal\_onset: [0.6759 3.9831e-18 5.3267e-14 0.3371 0.6785] |  |  |  |  |  |  |  |  |  |  |  |  |  |  |  |  |  |  |  |  |  |  |
| 63 |  | runProp\_onset: [0.0914 0.4198 0.4099 0.0519 0.0272] | nRun: 405 | pVal\_offset: [0.0199 0.2782 0.2020 0.0026 0.0244] |  |  |  |  |  |  |  |  |  |  |  |  |  |  |  |  |  |  |  |  |  |  |
| 64 |  | statProp\_offset: [0.5260 0.0617 0.0617 0.1461 0.2045] |  |  |  |  |  |  |  |  |  |  |  |  |  |  |  |  |  |  |  |  |  |  |  |  |
| 65 |  | runProp\_offset: [0.6123 0.0840 0.0889 0.0741 0.1407] |  |  |  |  |  |  |  |  |  |  |  |  |  |  |  |  |  |  |  |  |  |  |  |  |
| 66 |  |  |  |  |  |  |  |  |  |  |  |  |  |  |  |  |  |  |  |  |  |  |  |  |  |  |
| 67 | Sustainedness Index | Quantiles: top = stat, bottom = run |  | 1.0e-16 \* |  |  |  |  |  |  |  |  |  |  |  |  |  |  |  |  |  |  |  |  |  |  |
| 68 | 0 | 0.1198 0.2115 0.3377 | nBoth: 311 | 0.4935 |  |  |  |  |  |  |  |  |  |  |  |  |  |  |  |  |  |  |  |  |  |  |
| 69 |  | 0.2344 0.3855 0.5134 |  |  |  |  |  |  |  |  |  |  |  |  |  |  |  |  |  |  |  |  |  |  |  |  |
| 70 | 16 | 0.1987 0.3361 0.4998 | nBoth: 339 | 0.2928 |  |  |  |  |  |  |  |  |  |  |  |  |  |  |  |  |  |  |  |  |  |  |
| 71 |  | 0.3165 0.4800 0.6271 |  |  |  |  |  |  |  |  |  |  |  |  |  |  |  |  |  |  |  |  |  |  |  |  |
| 72 | 32 | 0.2047 0.3577 0.4876 | nBoth: 328 | 0 |  |  |  |  |  |  |  |  |  |  |  |  |  |  |  |  |  |  |  |  |  |  |
| 73 |  | 0.3535 0.5162 0.6473 |  |  |  |  |  |  |  |  |  |  |  |  |  |  |  |  |  |  |  |  |  |  |  |  |
| 74 | 64 | 0.2016 0.3587 0.5261 | nBoth: 309 | 0.9516 |  |  |  |  |  |  |  |  |  |  |  |  |  |  |  |  |  |  |  |  |  |  |
| 75 |  | 0.3609 0.5023 0.6249 |  |  |  |  |  |  |  |  |  |  |  |  |  |  |  |  |  |  |  |  |  |  |  |  |
| 76 | 128 | 0.2105 0.3632 0.5104 | nBoth: 266 | 0.0035 |  |  |  |  |  |  |  |  |  |  |  |  |  |  |  |  |  |  |  |  |  |  |
| 77 |  | 0.3606 0.5148 0.6430 |  |  |  |  |  |  |  |  |  |  |  |  |  |  |  |  |  |  |  |  |  |  |  |  |
| 78 | 256 | 0.2039 0.3541 0.4806 | nBoth: 254 | 0.1038 |  |  |  |  |  |  |  |  |  |  |  |  |  |  |  |  |  |  |  |  |  |  |
| 79 |  | 0.3482 0.5001 0.6054 |  |  |  |  |  |  |  |  |  |  |  |  |  |  |  |  |  |  |  |  |  |  |  |  |
| 80 |  |  |  |  |  |  |  |  |  |  |  |  |  |  |  |  |  |  |  |  |  |  |  |  |  |  |
| 81 |  |  |  |  |  |  |  |  |  |  |  |  |  |  |  |  |  |  |  |  |  |  |  |  |  |  |
| 82 |  |  |  |  |  |  |  |  |  |  |  |  |  |  |  |  |  |  |  |  |  |  |  |  |  |  |
| 83 | Cell types (Fig S2) |  |  |  |  |  |  |  |  |  |  |  |  |  |  |  |  |  |  |  |  |  |  |  |  |  |
| 84 | Response features | Proportions (Decay, Rise, Peak, Trough, Flat) | Binomial GLMEs as above, separate for each cell-type |  |  |  |  |  |  |  |  |  |  |  |  |  |  |  |  |  |  |  |  |  |  |  |
| 85 | Pyramidal | statProp\_onset: [0.1039 0.1419 0.6503 0.0390 0.0649] | # reliable responses | pVal\_onset: [0.3719 7.2090e-41 1.3987e-29 0.5967 0.1653] |  |  |  |  |  |  |  |  |  |  |  |  |  |  |  |  |  |  |  |  |  |  |
| 86 |  | runProp\_onset: [0.0929 0.4093 0.4115 0.0348 0.0515] | nStat: 1001 | pVal\_offset: [7.4961e-05 0.0880 0.6359 7.4715e-04 9.7084e-04] |  |  |  |  |  |  |  |  |  |  |  |  |  |  |  |  |  |  |  |  |  |  |
| 87 |  | statProp\_offset: [0.4625 0.0569 0.1009 0.0959 0.2837] | nRun: 1378 |  |  |  |  |  |  |  |  |  |  |  |  |  |  |  |  |  |  |  |  |  |  |  |
| 88 |  | runProp\_offset: [0.5450 0.0747 0.0951 0.0595 0.2257] |  |  |  |  |  |  |  |  |  |  |  |  |  |  |  |  |  |  |  |  |  |  |  |  |
| 89 |  |  |  |  |  |  |  |  |  |  |  |  |  |  |  |  |  |  |  |  |  |  |  |  |  |  |
| 90 | Narrow Interneurons | statProp\_onset: [0.0452 0.1036 0.7495 0.0640 0.0377] | nStat: 531 | pVal\_onset: [0.4061 3.1015e-33 1.1981e-24 0.0076 0.4231] |  |  |  |  |  |  |  |  |  |  |  |  |  |  |  |  |  |  |  |  |  |  |
| 91 |  | runProp\_onset: [0.0360 0.4453 0.4569 0.0322 0.0296] | nRun: 777 | pVal\_offset: [1.1451e-06 0.0886 0.8000 0.9661 3.7003e-10] |  |  |  |  |  |  |  |  |  |  |  |  |  |  |  |  |  |  |  |  |  |  |
| 92 |  | statProp\_offset: [0.4388 0.0433 0.0734 0.0734 0.3710] |  |  |  |  |  |  |  |  |  |  |  |  |  |  |  |  |  |  |  |  |  |  |  |  |
| 93 |  | runProp\_offset: [0.5663 0.0656 0.0772 0.0734 0.2175] |  |  |  |  |  |  |  |  |  |  |  |  |  |  |  |  |  |  |  |  |  |  |  |  |
| 94 |  |  |  |  |  |  |  |  |  |  |  |  |  |  |  |  |  |  |  |  |  |  |  |  |  |  |
| 95 | Wide Internuerons | statProp\_onset: [0.1064 0.1138 0.6881 0.0624 0.0294] | nStat: 545 | pVal\_onset: [0.4636 3.0446e-34 5.5207e-30 0.6826 0.5057] |  |  |  |  |  |  |  |  |  |  |  |  |  |  |  |  |  |  |  |  |  |  |
| 96 |  | runProp\_onset: [0.0943 0.4714 0.3416 0.0696 0.0232] | nRun: 647 | pVal\_offset: [0.0347 0.0714 2.2970e-04 0.0023 2.1178e-05] |  |  |  |  |  |  |  |  |  |  |  |  |  |  |  |  |  |  |  |  |  |  |
| 97 |  | statProp\_offset: [0.4807 0.0661 0.0569 0.2183 0.1780] |  |  |  |  |  |  |  |  |  |  |  |  |  |  |  |  |  |  |  |  |  |  |  |  |
| 98 |  | runProp\_offset: [0.5394 0.0958 0.1206 0.1515 0.0927] |  |  |  |  |  |  |  |  |  |  |  |  |  |  |  |  |  |  |  |  |  |  |  |  |
| 99 |  |  |  |  |  |  |  |  |  |  |  |  |  |  |  |  |  |  |  |  |  |  |  |  |  |  |
| 100 | Sustainedness Index | Quantiles: top = stat, bottom = run | LME, as above, separetly for cell-types |  |  |  |  |  |  |  |  |  |  |  |  |  |  |  |  |  |  |  |  |  |  |  |
| 101 | Pyramidal | susIndexQuant\_stat: [0.1924 0.3153 0.4752] | nBoth:844 | p = 2.2913e-50 |  |  |  |  |  |  |  |  |  |  |  |  |  |  |  |  |  |  |  |  |  |  |
| 102 |  | susIndexQuant\_run: [0.3146 0.4850 0.6197] |  |  |  |  |  |  |  |  |  |  |  |  |  |  |  |  |  |  |  |  |  |  |  |  |
| 103 |  |  |  |  |  |  |  |  |  |  |  |  |  |  |  |  |  |  |  |  |  |  |  |  |  |  |
| 104 | Narrow Interneurons | susIndexQuant\_stat: [0.1403 0.2985 0.4698] | nBoth: 480 | p =1.8159e-27 |  |  |  |  |  |  |  |  |  |  |  |  |  |  |  |  |  |  |  |  |  |  |
| 105 |  | susIndexQuant\_run: [0.3042 0.4510 0.5957] |  |  |  |  |  |  |  |  |  |  |  |  |  |  |  |  |  |  |  |  |  |  |  |  |
| 106 |  |  |  |  |  |  |  |  |  |  |  |  |  |  |  |  |  |  |  |  |  |  |  |  |  |  |
| 107 | Wide Internuerons | susIndexQuant\_stat: [0.2153 0.3623 0.5013] | nBoth: 483 | p = 1.1020e-29 |  |  |  |  |  |  |  |  |  |  |  |  |  |  |  |  |  |  |  |  |  |  |
| 108 |  | susIndexQuant\_run: [0.3579 0.5023 0.6363] |  |  |  |  |  |  |  |  |  |  |  |  |  |  |  |  |  |  |  |  |  |  |  |  |
| 109 |  |  |  |  |  |  |  |  |  |  |  |  |  |  |  |  |  |  |  |  |  |  |  |  |  |  |
| 110 |  |  |  |  |  |  |  |  |  |  |  |  |  |  |  |  |  |  |  |  |  |  |  |  |  |  |
| 111 |  |  |  |  |  |  |  |  |  |  |  |  |  |  |  |  |  |  |  |  |  |  |  |  |  |  |
| 112 |  |  |  |  |  |  |  |  |  |  |  |  |  |  |  |  |  |  |  |  |  |  |  |  |  |  |
| 113 | Visual areas (Fig S3) |  |  |  |  |  |  |  |  |  |  |  |  |  |  |  |  |  |  |  |  |  |  |  |  |  |
| 114 | Response features | Proportions (Decay, Rise, Peak, Trough, Flat) | Binomial GLMEs as above, separate for each speed |  |  |  |  |  |  |  |  |  |  |  |  |  |  |  |  |  |  |  |  |  |  |  |
| 115 | VISp | statProp\_onset: [0.1235 0.3019 0.4503 0.0620 0.0623] | # reliable responses (from n sesh) |  |  |  |  |  |  |  |  |  |  |  |  |  |  |  |  |  |  |  |  |  |  |  |
| 116 |  | runProp\_onset: [0.1233 0.4024 0.3467 0.0665 0.0611] | nStat: 3611 (13) | pVal\_onset: [0.6773 7.2633e-11 2.5590e-08 0.5902 0.8318] |  |  |  |  |  |  |  |  |  |  |  |  |  |  |  |  |  |  |  |  |  |  |
| 117 |  | statProp\_offset: [0.1235 0.3019 0.4503 0.0620 0.0623] | nRun: 3698 (11) | pVal\_offset: [0.1293 0.0073 0.0198 4.5861e-05 0.0154] |  |  |  |  |  |  |  |  |  |  |  |  |  |  |  |  |  |  |  |  |  |  |
| 118 |  | runProp\_offset: [0.1233 0.4024 0.3467 0.0665 0.0611] |  |  |  |  |  |  |  |  |  |  |  |  |  |  |  |  |  |  |  |  |  |  |  |  |
| 119 |  |  |  |  |  |  |  |  |  |  |  |  |  |  |  |  |  |  |  |  |  |  |  |  |  |  |
| 120 | VISl | statProp\_onset: [0.1463 0.2781 0.4113 0.0882 0.0760] | nStat: 3513 (11) | pVal\_onset: [0.9149 1.7852e-10 1.5883e-07 3.0080e-06 0.9085] |  |  |  |  |  |  |  |  |  |  |  |  |  |  |  |  |  |  |  |  |  |  |
| 121 |  | runProp\_onset: [0.1437 0.4929 0.2557 0.0344 0.0732] | nRun: 1830 (8) | pVal\_offset: [0.3187 0.1767 0.3108 1.0557e-07 0.9406] |  |  |  |  |  |  |  |  |  |  |  |  |  |  |  |  |  |  |  |  |  |  |
| 122 |  | statProp\_offset: [0.1463 0.2781 0.4113 0.0882 0.0760] |  |  |  |  |  |  |  |  |  |  |  |  |  |  |  |  |  |  |  |  |  |  |  |  |
| 123 |  | runProp\_offset: [0.1437 0.4929 0.2557 0.0344 0.0732] |  |  |  |  |  |  |  |  |  |  |  |  |  |  |  |  |  |  |  |  |  |  |  |  |
| 124 |  |  |  |  |  |  |  |  |  |  |  |  |  |  |  |  |  |  |  |  |  |  |  |  |  |  |
| 125 | VISal | statProp\_onset: [0.1261 0.3125 0.4060 0.0923 0.0631] | nStat: 1776 (11) | pVal\_onset: [0.1202 0.1161 0.0243 0.9438 0.3464] |  |  |  |  |  |  |  |  |  |  |  |  |  |  |  |  |  |  |  |  |  |  |
| 126 |  | runProp\_onset: [0.1874 0.3248 0.3208 0.0829 0.0841] | nRun: 3485 (9) | pVal\_offset: [3.6379e-04 0.2411 8.7358e-07 5.3875e-05 0.7041] |  |  |  |  |  |  |  |  |  |  |  |  |  |  |  |  |  |  |  |  |  |  |
| 127 |  | statProp\_offset: [0.1261 0.3125 0.4060 0.0923 0.0631] |  |  |  |  |  |  |  |  |  |  |  |  |  |  |  |  |  |  |  |  |  |  |  |  |
| 128 |  | runProp\_offset: [0.1874 0.3248 0.3208 0.0829 0.0841] |  |  |  |  |  |  |  |  |  |  |  |  |  |  |  |  |  |  |  |  |  |  |  |  |
| 129 |  |  |  |  |  |  |  |  |  |  |  |  |  |  |  |  |  |  |  |  |  |  |  |  |  |  |
| 130 | VISrl | statProp\_onset: [0.1581 0.3874 0.2940 0.0663 0.0942] | nStat: 1221 (11) | pVal\_onset: [0.7756 0.0031 0.1617 0.0685 0.4392] |  |  |  |  |  |  |  |  |  |  |  |  |  |  |  |  |  |  |  |  |  |  |
| 131 |  | runProp\_onset: [0.1658 0.4413 0.2467 0.0503 0.0960] | nRun: 1532 (11) | pVal\_offset: [0.0583 0.6608 0.0156 0.0028 0.1116] |  |  |  |  |  |  |  |  |  |  |  |  |  |  |  |  |  |  |  |  |  |  |
| 132 |  | statProp\_offset: [0.1581 0.3874 0.2940 0.0663 0.0942] |  |  |  |  |  |  |  |  |  |  |  |  |  |  |  |  |  |  |  |  |  |  |  |  |
| 133 |  | runProp\_offset: [0.1658 0.4413 0.2467 0.0503 0.0960] |  |  |  |  |  |  |  |  |  |  |  |  |  |  |  |  |  |  |  |  |  |  |  |  |
| 134 |  |  |  |  |  |  |  |  |  |  |  |  |  |  |  |  |  |  |  |  |  |  |  |  |  |  |
| 135 | VISam | statProp\_onset: [0.1267 0.3265 0.3840 0.0755 0.0873] | nStat: 2383 (14) | pVal\_onset: [0.7754 6.9551e-04 0.0753 0.0438 0.3652] |  |  |  |  |  |  |  |  |  |  |  |  |  |  |  |  |  |  |  |  |  |  |
| 136 |  | runProp\_onset: [0.1271 0.4105 0.3235 0.0558 0.0832] | nRun: 2597 (10) | pVal\_offset: [0.6530 0.9734 0.0043 1.7540e-08 0.2023] |  |  |  |  |  |  |  |  |  |  |  |  |  |  |  |  |  |  |  |  |  |  |
| 137 |  | statProp\_offset: [0.1267 0.3265 0.3840 0.0755 0.0873] |  |  |  |  |  |  |  |  |  |  |  |  |  |  |  |  |  |  |  |  |  |  |  |  |
| 138 |  | runProp\_offset: [0.1271 0.4105 0.3235 0.0558 0.0832] |  |  |  |  |  |  |  |  |  |  |  |  |  |  |  |  |  |  |  |  |  |  |  |  |
| 139 |  |  |  |  |  |  |  |  |  |  |  |  |  |  |  |  |  |  |  |  |  |  |  |  |  |  |
| 140 | VISpm | statProp\_onset: [0.0826 0.2860 0.4818 0.0826 0.0670] | nStat: 1731 (9) | pVal\_onset: [0.1504 0.0062 0.0160 0.7503 0.3942] |  |  |  |  |  |  |  |  |  |  |  |  |  |  |  |  |  |  |  |  |  |  |
| 141 |  | runProp\_onset: [0.1600 0.3754 0.2795 0.1018 0.0833] | nRun: 1356 (7) | pVal\_offset: [0.6385 5.5162e-05 0.0125 2.2659e-16 0.8762] |  |  |  |  |  |  |  |  |  |  |  |  |  |  |  |  |  |  |  |  |  |  |
| 142 |  | statProp\_offset: [0.0826 0.2860 0.4818 0.0826 0.0670] |  |  |  |  |  |  |  |  |  |  |  |  |  |  |  |  |  |  |  |  |  |  |  |  |
| 143 |  | runProp\_offset: [0.1600 0.3754 0.2795 0.1018 0.0833] |  |  |  |  |  |  |  |  |  |  |  |  |  |  |  |  |  |  |  |  |  |  |  |  |
| 144 |  |  |  |  |  |  |  |  |  |  |  |  |  |  |  |  |  |  |  |  |  |  |  |  |  |  |
| 145 | LGN | statProp\_onset: [0.1851 0.2645 0.3521 0.1091 0.0893] | nStat: 605 (4) | pVal\_onset: [0.7827 0.2583 0.0156 0.8497 0.8767] |  |  |  |  |  |  |  |  |  |  |  |  |  |  |  |  |  |  |  |  |  |  |
| 146 |  | runProp\_onset: [0.2261 0.3768 0.2283 0.0855 0.0832] | nRun: 889 (5) | pVal\_offset: [0.8813 0.8005 0.0520 0.1396 8.1621e-04] |  |  |  |  |  |  |  |  |  |  |  |  |  |  |  |  |  |  |  |  |  |  |
| 147 |  | statProp\_offset: [0.1851 0.2645 0.3521 0.1091 0.0893] |  |  |  |  |  |  |  |  |  |  |  |  |  |  |  |  |  |  |  |  |  |  |  |  |
| 148 |  | runProp\_offset: [0.2261 0.3768 0.2283 0.0855 0.0832] |  |  |  |  |  |  |  |  |  |  |  |  |  |  |  |  |  |  |  |  |  |  |  |  |
| 149 |  |  |  |  |  |  |  |  |  |  |  |  |  |  |  |  |  |  |  |  |  |  |  |  |  |  |
| 150 | LP | statProp\_onset: [0.2238 0.2885 0.2489 0.0874 0.1515] | nStat: 1591 (12) | pVal\_onset: [0.3870 0.0686 0.5577 0.9370 6.7671e-04] |  |  |  |  |  |  |  |  |  |  |  |  |  |  |  |  |  |  |  |  |  |  |
| 151 |  | runProp\_onset: [0.2047 0.4871 0.1647 0.0612 0.0824] | nRun: 850 (9) | pVal\_offset: [0.0190 0.3285 0.1012 0.1127 0.0080] |  |  |  |  |  |  |  |  |  |  |  |  |  |  |  |  |  |  |  |  |  |  |
| 152 |  | statProp\_offset: [0.2238 0.2885 0.2489 0.0874 0.1515] |  |  |  |  |  |  |  |  |  |  |  |  |  |  |  |  |  |  |  |  |  |  |  |  |
| 153 |  | runProp\_offset: [0.2047 0.4871 0.1647 0.0612 0.0824] |  |  |  |  |  |  |  |  |  |  |  |  |  |  |  |  |  |  |  |  |  |  |  |  |
| 154 |  |  |  |  |  |  |  |  |  |  |  |  |  |  |  |  |  |  |  |  |  |  |  |  |  |  |
| 155 |  |  |  |  |  |  |  |  |  |  |  |  |  |  |  |  |  |  |  |  |  |  |  |  |  |  |
| 156 |  |  |  |  |  |  |  |  |  |  |  |  |  |  |  |  |  |  |  |  |  |  |  |  |  |  |
| 157 | Pupil area (Fig S5) |  |  |  |  |  |  |  |  |  |  |  |  |  |  |  |  |  |  |  |  |  |  |  |  |  |
| 158 | Sustainedness index | Quantiles: top = small pupil, bottom = big pupil | # reliable responses |  |  |  |  |  |  |  |  |  |  |  |  |  |  |  |  |  |  |  |  |  |  |  |
| 159 |  | 0.2006 0.3288 0.4774 | nBoth = 1977 | p = 7.7341e-16 |  |  |  |  |  |  |  |  |  |  |  |  |  |  |  |  |  |  |  |  |  |  |
| 160 |  | 0.2207 0.3637 0.5120 |  |  |  |  |  |  |  |  |  |  |  |  |  |  |  |  |  |  |  |  |  |  |  |  |
| 161 |  |  |  |  |  |  |  |  |  |  |  |  |  |  |  |  |  |  |  |  |  |  |  |  |  |  |
| 162 | Response features |  |  |  |  |  |  |  |  |  |  |  |  |  |  |  |  |  |  |  |  |  |  |  |  |  |
| 163 |  | propSmallOn = 0.0843 0.1486 0.6492 0.0802 0.0378 |  | pVal\_onset = 0.0000 0.0020 0.0000 0.6480 0.8409 |  |  |  |  |  |  |  |  |  |  |  |  |  |  |  |  |  |  |  |  |  |  |
| 164 |  | propBigOn = 0.1216 0.1739 0.5895 0.0762 0.0389 |  | pVal\_offset = 0.0138 0.4145 0.3429 0.0000 0.0007 |  |  |  |  |  |  |  |  |  |  |  |  |  |  |  |  |  |  |  |  |  |  |
| 165 |  | propSmallOff = 0.4717 0.0739 0.0688 0.1246 0.2610 |  |  |  |  |  |  |  |  |  |  |  |  |  |  |  |  |  |  |  |  |  |  |  |  |
| 166 |  | propBigOff = 0.4363 0.0800 0.0758 0.1874 0.2205 |  |  |  |  |  |  |  |  |  |  |  |  |  |  |  |  |  |  |  |  |  |  |  |  |
| 167 |  |  |  |  |  |  |  |  |  |  |  |  |  |  |  |  |  |  |  |  |  |  |  |  |  |  |
| 168 |  |  | Compare big pupil to locomotion | pVal\_onset = 0.0000 0.0000 0.0000 0.0000 0.9940 |  |  |  |  |  |  |  |  |  |  |  |  |  |  |  |  |  |  |  |  |  |  |
| 169 |  |  |  | pVal\_offset = 0.0000 0.6879 0.0084 0.0000 0.0099 |  |  |  |  |  |  |  |  |  |  |  |  |  |  |  |  |  |  |  |  |  |  |
| 170 |  |  |  |  |  |  |  |  |  |  |  |  |  |  |  |  |  |  |  |  |  |  |  |  |  |  |
| 171 |  |  | Compare small pupil to locomotion | pVal\_onset = 0.3631 0.0000 0.0000 0.0000 0.8324 |  |  |  |  |  |  |  |  |  |  |  |  |  |  |  |  |  |  |  |  |  |  |
| 172 |  |  |  | pVal\_offset = 0.0000 0.6610 0.0005 0.0000 0.0000 |  |  |  |  |  |  |  |  |  |  |  |  |  |  |  |  |  |  |  |  |  |  |
