# Supplementary material for: Flexible neural population dynamics govern the speed and stability of sensory encoding in mouse visual cortex
Source: Nat Commun. 2024 Jul 30;15:6415. doi: 10.1038/s41467-024-50563-y (PMC11289260; doi:10.1038/s41467-024-50563-y)
Supplement: Supplementary file 8 — Source Data [file 41467_2024_50563_MOESM8_ESM.zip › TrajectoriesStats.html]

|  | A | B | C | D | E | F | G | H | I | J | K | L | M | N | O | P | Q | R | S | T | U | V | W | X | Y | Z | AA | AB |
| --- | --- | --- | --- | --- | --- | --- | --- | --- | --- | --- | --- | --- | --- | --- | --- | --- | --- | --- | --- | --- | --- | --- | --- | --- | --- | --- | --- | --- |
| 1 | Question/comparison | Values | Statistical test and n numbers | stat results |  |  |  |  |  |  |  |  |  |  |  |  |  |  |  |  |  |  |  |  |  |  |  |  |
| 2 |  |  |  |  |  |  |  |  |  |  |  |  |  |  |  |  |  |  |  |  |  |  |  |  |  |  |  |  |
| 3 | Distance measures [onset period] | Mean (SEM) | LME, n=5subs x 6 speeds |  |  |  |  |  |  |  |  |  |  |  |  |  |  |  |  |  |  |  |  |  |  |  |  |  |
| 4 |  |  | f = 'vals ~ state + (1|speed) + (1|sesh)'; |  |  |  |  |  |  |  |  |  |  |  |  |  |  |  |  |  |  |  |  |  |  |  |  |  |
| 5 | Distance travelled | Stat: 26.2406 (1.0390) |  | p = 0.22119 |  |  |  |  |  |  |  |  |  |  |  |  |  |  |  |  |  |  |  |  |  |  |  |  |
| 6 |  | Run: 25.1319 ( 1.0030) |  |  |  |  |  |  |  |  |  |  |  |  |  |  |  |  |  |  |  |  |  |  |  |  |  |  |
| 7 |  |  |  |  |  |  |  |  |  |  |  |  |  |  |  |  |  |  |  |  |  |  |  |  |  |  |  |  |
| 8 | Direct distance | Stat: 3.9880 ( 0.2903) |  | p = 9.3017e-19 |  |  |  |  |  |  |  |  |  |  |  |  |  |  |  |  |  |  |  |  |  |  |  |  |
| 9 |  | Run: 8.1150 (0.4322) |  |  |  |  |  |  |  |  |  |  |  |  |  |  |  |  |  |  |  |  |  |  |  |  |  |  |
| 10 |  |  |  |  |  |  |  |  |  |  |  |  |  |  |  |  |  |  |  |  |  |  |  |  |  |  |  |  |
| 11 | Distance Ratio (stats on log2 transform) | Stat: 7.8033 (0.8106) |  | p = 3.7498e-19 |  |  |  |  |  |  |  |  |  |  |  |  |  |  |  |  |  |  |  |  |  |  |  |  |
| 12 |  | Run: 3.2266 (0.1308) |  |  |  |  |  |  |  |  |  |  |  |  |  |  |  |  |  |  |  |  |  |  |  |  |  |  |
| 13 |  |  |  |  |  |  |  |  |  |  |  |  |  |  |  |  |  |  |  |  |  |  |  |  |  |  |  |  |
| 14 |  |  |  |  |  |  |  |  |  |  |  |  |  |  |  |  |  |  |  |  |  |  |  |  |  |  |  |  |
| 15 | Distance measures [offset period] | Mean (SEM) | LME, n=5subs x 6 speeds |  |  |  |  |  |  |  |  |  |  |  |  |  |  |  |  |  |  |  |  |  |  |  |  |  |
| 16 |  |  | f = 'vals ~ state + (1|speed) + (1|sesh)'; |  |  |  |  |  |  |  |  |  |  |  |  |  |  |  |  |  |  |  |  |  |  |  |  |  |
| 17 | Distance travelled | 3.4389 0.2655 |  | p = 2.9479e-10 |  |  |  |  |  |  |  |  |  |  |  |  |  |  |  |  |  |  |  |  |  |  |  |  |
| 18 |  | 5.2039 0.3384 |  |  |  |  |  |  |  |  |  |  |  |  |  |  |  |  |  |  |  |  |  |  |  |  |  |  |
| 19 |  |  |  |  |  |  |  |  |  |  |  |  |  |  |  |  |  |  |  |  |  |  |  |  |  |  |  |  |
| 20 | Direct distance | 1.8828 0.1703 |  | p = 1.6257E-17 |  |  |  |  |  |  |  |  |  |  |  |  |  |  |  |  |  |  |  |  |  |  |  |  |
| 21 |  | 3.7855 0.2412 |  |  |  |  |  |  |  |  |  |  |  |  |  |  |  |  |  |  |  |  |  |  |  |  |  |  |
| 22 |  |  |  |  |  |  |  |  |  |  |  |  |  |  |  |  |  |  |  |  |  |  |  |  |  |  |  |  |
| 23 | Distance Ratio | 2.0462 0.1440 |  | p = 2.7651e-07 |  |  |  |  |  |  |  |  |  |  |  |  |  |  |  |  |  |  |  |  |  |  |  |  |
| 24 |  | 1.3763 0.0309 |  |  |  |  |  |  |  |  |  |  |  |  |  |  |  |  |  |  |  |  |  |  |  |  |  |  |
| 25 |  |  |  |  |  |  |  |  |  |  |  |  |  |  |  |  |  |  |  |  |  |  |  |  |  |  |  |  |
| 26 |  |  |  |  |  |  |  |  |  |  |  |  |  |  |  |  |  |  |  |  |  |  |  |  |  |  |  |  |
| 27 |  |  |  |  |  |  |  |  |  |  |  |  |  |  |  |  |  |  |  |  |  |  |  |  |  |  |  |  |
| 28 | Speed and Acceleration | Mean (SEM) |  |  |  |  |  |  |  |  |  |  |  |  |  |  |  |  |  |  |  |  |  |  |  |  |  |  |
| 29 | Max speed (onset) | Stat: 8.5380 (0.4404) |  | p =2.7519e-07 |  |  |  |  |  |  |  |  |  |  |  |  |  |  |  |  |  |  |  |  |  |  |  |  |
| 30 |  | Run: 10.3263 ( 0.3659) |  |  |  |  |  |  |  |  |  |  |  |  |  |  |  |  |  |  |  |  |  |  |  |  |  |  |
| 31 |  |  |  |  |  |  |  |  |  |  |  |  |  |  |  |  |  |  |  |  |  |  |  |  |  |  |  |  |
| 32 | Offset | stat: 4.1932 (0.3619) |  | p = 1.1402e-10 |  |  |  |  |  |  |  |  |  |  |  |  |  |  |  |  |  |  |  |  |  |  |  |  |
| 33 |  | run: 6.3788 ( 0.4273) |  |  |  |  |  |  |  |  |  |  |  |  |  |  |  |  |  |  |  |  |  |  |  |  |  |  |
| 34 |  |  |  |  |  |  |  |  |  |  |  |  |  |  |  |  |  |  |  |  |  |  |  |  |  |  |  |  |
| 35 | Relative spectral power of accelerations | Mean (SEM) |  |  |  |  |  |  |  |  |  |  |  |  |  |  |  |  |  |  |  |  |  |  |  |  |  |  |
| 36 | Low band (0-6 Hz) | Stat: 0.9829 ( 0.0842) |  | adtest for normality, p = 0.4543 |  |  |  |  |  |  |  |  |  |  |  |  |  |  |  |  |  |  |  |  |  |  |  |  |
| 37 | power spectrum (0-20Hz) normalised by mean of spectrum | Run: 1.6003 (0.0505) | paired t-test | p = 7.4565e-07 |  |  |  |  |  |  |  |  |  |  |  |  |  |  |  |  |  |  |  |  |  |  |  |  |
| 38 |  |  |  |  |  |  |  |  |  |  |  |  |  |  |  |  |  |  |  |  |  |  |  |  |  |  |  |  |
| 39 | High band (6-20 Hz) | Stat: 1.0068 (0.0401) |  | same p-values since using relative power |  |  |  |  |  |  |  |  |  |  |  |  |  |  |  |  |  |  |  |  |  |  |  |  |
| 40 |  | Run: 0.7153 (0.0240) | paired t-test |  |  |  |  |  |  |  |  |  |  |  |  |  |  |  |  |  |  |  |  |  |  |  |  |  |
| 41 |  |  |  |  |  |  |  |  |  |  |  |  |  |  |  |  |  |  |  |  |  |  |  |  |  |  |  |  |
| 42 |  |  |  |  |  |  |  |  |  |  |  |  |  |  |  |  |  |  |  |  |  |  |  |  |  |  |  |  |
| 43 | Angle of approach | Mean (SEM) | LME |  |  |  |  |  |  |  |  |  |  |  |  |  |  |  |  |  |  |  |  |  |  |  |  |  |
| 44 | Cumulative change in angle (onset) | Stat: 516.9536 (18.1672)  Run: 318.8556 (9.4587) | f = 'vals ~ state + (1|speed) + (1|sesh)'; | p = 2.5761e-17 |  |  |  |  |  |  |  |  |  |  |  |  |  |  |  |  |  |  |  |  |  |  |  |  |
| 45 |  |  |  |  |  |  |  |  |  |  |  |  |  |  |  |  |  |  |  |  |  |  |  |  |  |  |  |  |
| 46 | Offset | Stat: 363.2068 (15.9508) |  | p = 9.4107e-13; |  |  |  |  |  |  |  |  |  |  |  |  |  |  |  |  |  |  |  |  |  |  |  |  |
| 47 |  | Run: 220.9804 (11.8478) |  |  |  |  |  |  |  |  |  |  |  |  |  |  |  |  |  |  |  |  |  |  |  |  |  |  |
| 48 |  |  |  |  |  |  |  |  |  |  |  |  |  |  |  |  |  |  |  |  |  |  |  |  |  |  |  |  |
| 49 |  |  |  |  |  |  |  |  |  |  |  |  |  |  |  |  |  |  |  |  |  |  |  |  |  |  |  |  |
| 50 | Trajectory tangling |  | RM-ANOVA (n=5subs x 6 speeds) |  |  |  |  |  |  |  |  |  |  |  |  |  |  |  |  |  |  |  |  |  |  |  |  |  |
| 51 |  |  | [p,tbl,stats,terms] = anovan(allVals,{timeVec,stateVec,subjVec},'model',2,'random',3,'varnames',{'Time','State','Subj'}); |  |  |  |  |  |  |  |  |  |  |  |  |  |  |  |  |  |  |  |  |  |  |  |  |  |
| 52 |  |  |  | Source Sum Sq. d.f. Mean Sq. F Prob>F -----------------------------------------------------------  Time 0.00622 179 0.00003 3.35 0   State 0.00698 1 0.00698 18.91 0.0025  Subj 0.00295 4 0.00074 1.7 0.3063  Time\*State 0.00537 179 0.00003 8.36 0   Time\*Subj 0.00743 716 0.00001 2.9 0   State\*Subj 0.00171 4 0.00043 119.21 0   Error 0.03482 9716 0   Total 0.06548 10799 |  |  |  |  |  |  |  |  |  |  |  |  |  |  |  |  |  |  |  |  |  |  |  |  |
| 53 |  |  |  |  |  |  |  |  |  |  |  |  |  |  |  |  |  |  |  |  |  |  |  |  |  |  |  |  |
| 54 | Distance between trajectories | Mean (SEM) | paired t-test on mean range over stimulus perirod | [h,p] = adtest(meanStat-meanRun), p = 0.1725 |  |  |  |  |  |  |  |  |  |  |  |  |  |  |  |  |  |  |  |  |  |  |  |  |
| 55 |  | Stat: 2.8697 (0.4900) | n = 5 subs | ttest, p = 1.5966e-04 |  |  |  |  |  |  |  |  |  |  |  |  |  |  |  |  |  |  |  |  |  |  |  |  |
| 56 |  | Run: 5.9521 (0.5448) |  |  |  |  |  |  |  |  |  |  |  |  |  |  |  |  |  |  |  |  |  |  |  |  |  |  |
| 57 |  |  |  |  |  |  |  |  |  |  |  |  |  |  |  |  |  |  |  |  |  |  |  |  |  |  |  |  |
| 58 | LDA decoding | RM ANOVA | [p,tbl,stats,terms] = anovan(decVec,{timeVec,stateVec,subjVec},'model',2,'random',3,'varnames',{'Time','State','Subj'}); | ----------------------------------------------------------  Source Sum Sq. d.f. Mean Sq. F Prob>F ----------------------------------------------------------  Time 56.0652 189 0.2966 85.95 0   State 11.0198 1 11.0198 37.86 0.0035  Subj 4.3994 4 1.0999 3.77 0.1129  Time\*State 1.9574 189 0.0104 3.61 0   Time\*Subj 2.6091 756 0.0035 1.2 0.0056  State\*Subj 1.1642 4 0.291 101.45 0   Error 2.1689 756 0.0029   Total 79.3841 1899 |  |  |  |  |  |  |  |  |  |  |  |  |  |  |  |  |  |  |  |  |  |  |  |  |
| 59 |  |  |  |  |  |  |  |  |  |  |  |  |  |  |  |  |  |  |  |  |  |  |  |  |  |  |  |  |
| 60 |  |  |  |  |  |  |  |  |  |  |  |  |  |  |  |  |  |  |  |  |  |  |  |  |  |  |  |  |
| 61 | Cross-condiiton tangling | RM ANOVAa | [p,tbl,stats,terms] = anovan(allVals,{timeVec,stateVec,subjVec},'model',2,'random',3,'varnames',{'Time','State','Subj'}); | ----------------------------------------------------------  Source Sum Sq. d.f. Mean Sq. F Prob>F -----------------------------------------------------------  Time 0.0133 179 0.00007 80.56 0   State 0.00398 1 0.00398 121.06 0   Subj 0.00015 4 0.00004 2.24 0.305   Time\*State 0.00274 179 0.00002 28.74 0   Time\*Subj 0.00066 716 0 1.72 0   State\*Subj 0.00009 4 0.00002 4.3 0.3286  Error 0.02822 52916 0   Total 0.04915 53999 |  |  |  |  |  |  |  |  |  |  |  |  |  |  |  |  |  |  |  |  |  |  |  |  |
| 62 |  |  |  |  |  |  |  |  |  |  |  |  |  |  |  |  |  |  |  |  |  |  |  |  |  |  |  |  |
| 63 |  |  |  |  |  |  |  |  |  |  |  |  |  |  |  |  |  |  |  |  |  |  |  |  |  |  |  |  |
| 64 |  |  |  |  |  |  |  |  |  |  |  |  |  |  |  |  |  |  |  |  |  |  |  |  |  |  |  |  |
| 65 |  |  |  |  |  |  |  |  |  |  |  |  |  |  |  |  |  |  |  |  |  |  |  |  |  |  |  |  |
| 66 |  |  |  |  |  |  |  |  |  |  |  |  |  |  |  |  |  |  |  |  |  |  |  |  |  |  |  |  |
| 67 | Single trial analyses |  |  |  |  |  |  |  |  |  |  |  |  |  |  |  |  |  |  |  |  |  |  |  |  |  |  |  |
| 68 |  |  |  |  |  |  |  |  |  |  |  |  |  |  |  |  |  |  |  |  |  |  |  |  |  |  |  |  |
| 69 | Distance ratios | Mean (SEM) | LME |  |  |  |  |  |  |  |  |  |  |  |  |  |  |  |  |  |  |  |  |  |  |  |  |  |
| 70 | Onset | Stat: 5.9616 (0.2457) | f = 'vals ~ state + (1|speed) + (1|sesh)'; | p = 3.7556e-17 |  |  |  |  |  |  |  |  |  |  |  |  |  |  |  |  |  |  |  |  |  |  |  |  |
| 71 |  | Run: 3.5406 (0.1175) |  |  |  |  |  |  |  |  |  |  |  |  |  |  |  |  |  |  |  |  |  |  |  |  |  |  |
| 72 |  |  |  |  |  |  |  |  |  |  |  |  |  |  |  |  |  |  |  |  |  |  |  |  |  |  |  |  |
| 73 | Offset | Stat 3.3985 (0.2760) |  | p = 0.0026599 |  |  |  |  |  |  |  |  |  |  |  |  |  |  |  |  |  |  |  |  |  |  |  |  |
| 74 |  | Run: 2.6915 (0.0603) |  |  |  |  |  |  |  |  |  |  |  |  |  |  |  |  |  |  |  |  |  |  |  |  |  |  |
| 75 |  |  |  |  |  |  |  |  |  |  |  |  |  |  |  |  |  |  |  |  |  |  |  |  |  |  |  |  |
| 76 |  |  |  |  |  |  |  |  |  |  |  |  |  |  |  |  |  |  |  |  |  |  |  |  |  |  |  |  |
| 77 |  |  |  |  |  |  |  |  |  |  |  |  |  |  |  |  |  |  |  |  |  |  |  |  |  |  |  |  |
| 78 |  |  |  |  |  |  |  |  |  |  |  |  |  |  |  |  |  |  |  |  |  |  |  |  |  |  |  |  |
| 79 | Trajectory tangling |  | RM-ANOVA, n =5 subs x 6 speeds (single-trial tanngling averaged to give conditon mean) |  |  |  |  |  |  |  |  |  |  |  |  |  |  |  |  |  |  |  |  |  |  |  |  |  |
| 80 |  |  | [p,tbl,stats,terms] = anovan(allVals,{timeVec,stateVec,subjVec,speedVec},'model','full','varnames',{'Time','State','Subj','Speed'}); |  |  |  |  |  |  |  |  |  |  |  |  |  |  |  |  |  |  |  |  |  |  |  |  |  |
| 81 |  |  |  |  |  |  |  |  |  |  |  |  |  |  |  |  |  |  |  |  |  |  |  |  |  |  |  |  |
| 82 |  |  |  |  |  |  |  |  |  |  |  |  |  |  |  |  |  |  |  |  |  |  |  |  |  |  |  |  |
| 83 |  |  |  |  |  |  |  |  |  |  |  |  |  |  |  |  |  |  |  |  |  |  |  |  |  |  |  |  |
| 84 |  |  |  |  |  |  |  |  |  |  |  |  |  |  |  |  |  |  |  |  |  |  |  |  |  |  |  |  |
| 85 |  |  |  |  |  |  |  |  |  |  |  |  |  |  |  |  |  |  |  |  |  |  |  |  |  |  |  |  |
| 86 | Max traj speed |  | LME on top speed |  |  |  |  |  |  |  |  |  |  |  |  |  |  |  |  |  |  |  |  |  |  |  |  |  |
| 87 | Onset | Mean (SEM) | f = 'vals ~ state + (1|speed) + (1|sesh)'; | p = 2.2739e-91 |  |  |  |  |  |  |  |  |  |  |  |  |  |  |  |  |  |  |  |  |  |  |  |  |
| 88 |  | Stat: 5.5875 (0.1275) |  |  |  |  |  |  |  |  |  |  |  |  |  |  |  |  |  |  |  |  |  |  |  |  |  |  |
| 89 |  | Run: 5.0372 (0.1479) |  |  |  |  |  |  |  |  |  |  |  |  |  |  |  |  |  |  |  |  |  |  |  |  |  |  |
| 90 |  |  |  |  |  |  |  |  |  |  |  |  |  |  |  |  |  |  |  |  |  |  |  |  |  |  |  |  |
| 91 | Offset | Stat: 3.0462 (0.1466) |  | p = 6.5038E-64 |  |  |  |  |  |  |  |  |  |  |  |  |  |  |  |  |  |  |  |  |  |  |  |  |
| 92 |  | Run: 3.6087 (0.0873) |  |  |  |  |  |  |  |  |  |  |  |  |  |  |  |  |  |  |  |  |  |  |  |  |  |  |
| 93 |  |  |  |  |  |  |  |  |  |  |  |  |  |  |  |  |  |  |  |  |  |  |  |  |  |  |  |  |
| 94 | Power spectrum of accelerations -> can't do on single trials (too noisy) |  |  |  |  |  |  |  |  |  |  |  |  |  |  |  |  |  |  |  |  |  |  |  |  |  |  |  |
| 95 |  |  |  |  |  |  |  |  |  |  |  |  |  |  |  |  |  |  |  |  |  |  |  |  |  |  |  |  |
| 96 |  |  |  |  |  |  |  |  |  |  |  |  |  |  |  |  |  |  |  |  |  |  |  |  |  |  |  |  |
| 97 |  |  |  |  |  |  |  |  |  |  |  |  |  |  |  |  |  |  |  |  |  |  |  |  |  |  |  |  |
| 98 | Angle of approach | Mean (SEM) |  |  |  |  |  |  |  |  |  |  |  |  |  |  |  |  |  |  |  |  |  |  |  |  |  |  |
| 99 | Onset | Stat: 570.2476 (24.5051) | f = 'vals ~ state + (1|speed) + (1|sesh)'; | p = 1.0438e-96 |  |  |  |  |  |  |  |  |  |  |  |  |  |  |  |  |  |  |  |  |  |  |  |  |
| 100 |  | Run: 524.2078 (26.0895) |  |  |  |  |  |  |  |  |  |  |  |  |  |  |  |  |  |  |  |  |  |  |  |  |  |  |
| 101 |  |  |  |  |  |  |  |  |  |  |  |  |  |  |  |  |  |  |  |  |  |  |  |  |  |  |  |  |
| 102 | Offset | Stat: 495.3645 (28.6612) |  | p = 1.4504e-152 |  |  |  |  |  |  |  |  |  |  |  |  |  |  |  |  |  |  |  |  |  |  |  |  |
| 103 |  | Run: 415.1111 (19.0039) |  |  |  |  |  |  |  |  |  |  |  |  |  |  |  |  |  |  |  |  |  |  |  |  |  |  |
