# Supplementary material for: Flexible neural population dynamics govern the speed and stability of sensory encoding in mouse visual cortex
Source: Nat Commun. 2024 Jul 30;15:6415. doi: 10.1038/s41467-024-50563-y (PMC11289260; doi:10.1038/s41467-024-50563-y)
Supplement: Supplementary file 8 — Source Data [file 41467_2024_50563_MOESM8_ESM.zip › TuningStats.html]

|  | A | B | C | D | E | F | G | H | I | J | K | L | M | N | O | P | Q | R | S | T | U | V | W | X | Y | Z |
| --- | --- | --- | --- | --- | --- | --- | --- | --- | --- | --- | --- | --- | --- | --- | --- | --- | --- | --- | --- | --- | --- | --- | --- | --- | --- | --- |
| 1 | Question/comparison | Values | Statistical test and n numbers | stat results |  |  |  |  |  |  |  |  |  |  |  |  |  |  |  |  |  |  |  |  |  |  |
| 2 | Tuning bouts |  | McNemar |  |  |  |  |  |  |  |  |  |  |  |  |  |  |  |  |  |  |  |  |  |  |  |
| 3 | Are more units tuned during locomotion? |  | total N = 1583 |  |  |  |  |  |  |  |  |  |  |  |  |  |  |  |  |  |  |  |  |  |  |  |
| 4 |  | StatGood: 407 (25.71%) RunGood: 606 (38.28%) BothGood:344 (21.73%) | (+stat+run, +stat-run  -stat+run, -stat-run) |  |  |  |  |  |  |  |  |  |  |  |  |  |  |  |  |  |  |  |  |  |  |  |
| 5 |  |  | 344 63 262 914 |  |  |  |  |  |  |  |  |  |  |  |  |  |  |  |  |  |  |  |  |  |  |  |
| 6 |  |  |  |  |  |  |  |  |  |  |  |  |  |  |  |  |  |  |  |  |  |  |  |  |  |  |
| 7 |  |  |  |  |  |  |  |  |  |  |  |  |  |  |  |  |  |  |  |  |  |  |  |  |  |  |
| 8 | Tuning start times | Quantiles | LME |  |  |  |  |  |  |  |  |  |  |  |  |  |  |  |  |  |  |  |  |  |  |  |
| 9 |  | Stat: 0.0600 0.1700 0.4400 | f = 'startTime ~ state + (1|unit) + (1|sesh)'; | p = 3.3555E-11 |  |  |  |  |  |  |  |  |  |  |  |  |  |  |  |  |  |  |  |  |  |  |
| 10 |  | Run: 0.0500 0.1000 0.2100 | n = 688 tuned in both |  |  |  |  |  |  |  |  |  |  |  |  |  |  |  |  |  |  |  |  |  |  |  |
| 11 |  |  |  |  |  |  |  |  |  |  |  |  |  |  |  |  |  |  |  |  |  |  |  |  |  |  |
| 12 | Tuning finish times | Quantiles | LME |  |  |  |  |  |  |  |  |  |  |  |  |  |  |  |  |  |  |  |  |  |  |  |
| 13 |  | Stat: 0.8250 1.0700 1.1650 | f = 'FinishTime ~ state + (1|unit) + (1|sesh)'; | p = 1.645e-13 |  |  |  |  |  |  |  |  |  |  |  |  |  |  |  |  |  |  |  |  |  |  |
| 14 |  | Run: 1.0500 1.1500 1.3400 | n = 688 tuned in both |  |  |  |  |  |  |  |  |  |  |  |  |  |  |  |  |  |  |  |  |  |  |  |
| 15 |  |  |  |  |  |  |  |  |  |  |  |  |  |  |  |  |  |  |  |  |  |  |  |  |  |  |
| 16 | Tuning durations | Quantiles | LME |  |  |  |  |  |  |  |  |  |  |  |  |  |  |  |  |  |  |  |  |  |  |  |
| 17 |  | Stat: 0.1800 0.4650 0.9000 | f = Duration ~ state + (1|unit) + (1|sesh)'; | p =6.4217e-46 |  |  |  |  |  |  |  |  |  |  |  |  |  |  |  |  |  |  |  |  |  |  |
| 18 |  | Run: 0.5350 0.9550 1.1300 | n = 688 tuned in both |  |  |  |  |  |  |  |  |  |  |  |  |  |  |  |  |  |  |  |  |  |  |  |
| 19 |  |  |  |  |  |  |  |  |  |  |  |  |  |  |  |  |  |  |  |  |  |  |  |  |  |  |
| 20 |  |  |  |  |  |  |  |  |  |  |  |  |  |  |  |  |  |  |  |  |  |  |  |  |  |  |
| 21 | Dynamic range | Mean (SEM) | LME |  |  |  |  |  |  |  |  |  |  |  |  |  |  |  |  |  |  |  |  |  |  |  |
| 22 | Mean value over stimulus period | Stat: 2.1741 (0.1093) | f = 'vals ~ state + (1|unit) + (1|sesh)'; | p = 1.4188e-76 |  |  |  |  |  |  |  |  |  |  |  |  |  |  |  |  |  |  |  |  |  |  |
| 23 |  | Run: 4.4138 (0.1917) | n=1,583 good units |  |  |  |  |  |  |  |  |  |  |  |  |  |  |  |  |  |  |  |  |  |  |  |
| 24 |  |  |  |  |  |  |  |  |  |  |  |  |  |  |  |  |  |  |  |  |  |  |  |  |  |  |
| 25 |  |  |  |  |  |  |  |  |  |  |  |  |  |  |  |  |  |  |  |  |  |  |  |  |  |  |
| 26 |  |  |  |  |  |  |  |  |  |  |  |  |  |  |  |  |  |  |  |  |  |  |  |  |  |  |
| 27 |  |  |  |  |  |  |  |  |  |  |  |  |  |  |  |  |  |  |  |  |  |  |  |  |  |  |
| 28 |  |  |  |  |  |  |  |  |  |  |  |  |  |  |  |  |  |  |  |  |  |  |  |  |  |  |
| 29 |  |  |  |  |  |  |  |  |  |  |  |  |  |  |  |  |  |  |  |  |  |  |  |  |  |  |
| 30 |  |  |  |  |  |  |  |  |  |  |  |  |  |  |  |  |  |  |  |  |  |  |  |  |  |  |
| 31 | LDA - indy neurons by session | RM-ANOVA | [p,tbl,stats,terms] = anovan(decVec,{timeVec,stateVec,subjVec},'model',2,'random',3,'varnames',{'Time','State','Subj'}); |  |  |  |  |  |  |  |  |  |  |  |  |  |  |  |  |  |  |  |  |  |  |  |
| 32 |  | ---------------------------------------------------------- |  |  |  |  |  |  |  |  |  |  |  |  |  |  |  |  |  |  |  |  |  |  |  |  |
| 33 |  | Source Sum Sq. d.f. Mean Sq. F Prob>F ----------------------------------------------------------  Time 47.1951 190 0.2484 67.1 0   State 12.4225 1 12.4225 183.15 0.0002  Subj 4.8857 4 1.2214 17.64 0.0072  Time\*State 3.6898 190 0.0194 8.52 0   Time\*Subj 2.8132 760 0.0037 1.62 0   State\*Subj 0.2713 4 0.0678 29.77 0   Error 1.7318 760 0.0023   Total 73.0094 1909 | n = 5 subjects |  |  |  |  |  |  |  |  |  |  |  |  |  |  |  |  |  |  |  |  |  |  |  |
